# The effect of universal school-based mindfulness on anhedonia and emotional distress and its underlying mechanisms: A cluster randomised controlled trial via experience sampling in secondary schools

**DOI:** 10.1016/j.brat.2023.104405

**Published:** 2023-10

**Authors:** Liesbeth Bogaert, Katleen Van der Gucht, Peter Kuppens, Merle Kock, Marieke J. Schreuder, Willem Kuyken, Filip Raes

**Affiliations:** a Research Unit Behaviour, Health and Psychopathology, KU Leuven, Belgium; b Leuven Mindfulness Centre, KU Leuven, Belgium; c KU Leuven Child and Youth Institute, KU Leuven, Belgium; d Research Unit Methods, Individual and Cultural Differences, Affect and Social Behavior, KU Leuven, Belgium; e Social and Behavioral Sciences, Tilburg University, the Netherlands; f Department of Psychiatry, Medical Sciences Division, University of Oxford, United Kingdom; g University of Oxford Mindfulness Research Centre, University of Oxford, United Kingdom

**Keywords:** Mindfulness, Adolescents, Emotional distress, Anhedonia, Schools

## Abstract

This cluster randomised controlled trial examined the effectiveness of universal school-based mindfulness training (MT; vs. passive control) to lower anhedonia and emotional distress among mid-adolescents (15–18 years). It further examined three potential mechanisms: dampening of positive emotions, non-acceptance/suppression of negative emotions, and perceived social pressure not to experience/express negative emotions. Adolescents (*n*_control_ = 136, *n*_intervention_ = 95) participated in three assessment points (before, after and two/three months after the in-class MT), consisting of Experience Sampling (ES) assessments and self-report questionnaires (SRQs) to corroborate the ES assessments. Analyses were based on general linear modelling and multilevel modelling. Overall, no evidence was found for a significant beneficial and long-lasting impact of the MT on adolescents' mental health. Importantly, some barriers inherently linked to universal MT approaches (low engagement in and mixed attitudes towards the MT) may have tempered the effectiveness of the MT in the current trial. Further research should prioritise overcoming these barriers to optimise programme implementation. Additionally, given the potential complex interplay of moderators at micro- (home practice), meso- (school climate), and macro-level (broader context), research should simultaneously focus on alternative ways of delivering MT at schools to strengthen adolescents’ mental health.

## Highlights

• Does adolescents' mental health benefit from universal school-based mindfulness?
• Current *c*-RCT based on experience sampling methodology yielded no evidence.
• No improvement in mental health measures or expected underlying mechanisms.
• Further research on universal approach should prioritise focus on moderators.
• Focus on alternative ways to implement mindfulness at school might be fruitful.

Among adolescents, mental health problems have been identified as a major leading cause of disability worldwide and over the years a rising trend has been observed (Akseer et al., 2020; Armocida et al., 2022; Mojtabai, Olfson, & Han, 2016). Mental health problems place an enormous burden on adolescents, with depression being the leading cause of years lost due to disability (GBD 2019 Mental Disorders Collaborators, 2022). All too often depressive symptoms persist, prevent adolescents from thriving in multiple life domains (e.g., social and occupational impairment) and can lead to long-term severe psychiatric problems (Avenevoli, Swendsen, He, Burstein, & Merikangas, 2015; Birmaher, Arbelaez, & Brent, 2002; Patel, Flisher, Hetrick, & McGorry, 2007). Together these findings are a call to action for prevention of and early intervention for mental ill-health among adolescents.

One promising answer to this call are universal school-based approaches. Compared to targeted approaches, universal programmes can reach and benefit large groups of adolescents regardless of risk (Greenberg, Domitrovich, Weissberg, & Durlak, 2017; Durlak et al., 2022; Werner-Seidler, Perry, Calear, Newby, & Christensen, 2017). Universal approaches do not single out and therefore stigmatize selected adolescents (Foulkes & Stapley, 2022). Furthermore, school settings are particularly suitable for the implementation of universal programmes due to their broach reach and centrality in the lives of adolescents (Greenberg & Abenavoli, 2016). Finally, there is promising evidence of the effectiveness of school-based universal programmes on several mental health related outcomes (Cefai et al., 2022; O’Conner, Dyson, Cowdell, & Watson, 2017; Werner-Seidler et al., 2017). One particular programme that has attracted researchers' attention over the years is school-based Mindfulness Training (MT).

Mindfulness refers to a compassionate and non-judgmental moment to moment awareness of one's experiences (Kabat-Zinn, 1994, p. 4). Two common structured interventions to teach mindfulness skills are Mindfulness-Based Stress Reduction (MBSR; Kabat-Zinn, 1990) and Mindfulness-Based Cognitive Therapy (MBCT; Segal, Williams, & Teasdale, 2002). Participants in mindfulness-based programmes learn the capacity to be non-judgmentally aware of thoughts, feelings and sensations, and to replace automatic, habitual, and often judgmental reactions with more conscious and skilful responses (Van der Gucht, 2017). In adults, MT has been proven effective in boosting well-being and ameliorating mood disturbances in a wide range of conditions, in both treatment and prevention contexts (Goyal et al., 2014; Khoury et al., 2013, 2015). In younger age groups, overall and across contexts, MT has been shown to have similar beneficial effects (Reangsing, Punsuwun, & Sneider, 2021).

Concerning universal school-based MT in particular, while a large body of work suggests the positive impact on mental health and well-being of adolescents (e.g., Baelen, Esposito, & Galla, 2019; Carsley, Khoury, & Heath, 2018; Dunning et al., 2019; Raes, Griffith, Van der Gucht, & Williams, 2014; Roeser, Galla, & Baelen, 2020), there are also studies showing no effects (e.g., Johnson, Burke, Brinkman, & Wade, 2016, 2017; Volanen et al., 2020). This absence of conclusive evidence is reflected in recently published reviews concluding that the overall effects of school-based (targeted and universal) mindfulness interventions in terms of clinical utility is not convincing (Clark et al., 2021; Fulambarkar et al., 2022; Philips & Mychailyszyn, 2022; Tudor et al., 2022). Overall, rather small effect sizes are found which often become insignificant when only considering studies including active control groups (Fulambarkar et al., 2022; Philips & Mychailyszyn, 2022).

The more recently published MYRIAD trial was a fully powered, rigorous cluster randomised controlled trial (RCT) evaluating the effectiveness of universal school-based MT in early adolescence on mental health and well-being (Kuyken et al., 2022). This trial did not yield support for the superiority of universal school-based MT compared to normal provision of social-emotional education (Kuyken et al., 2022), which further tempers the overall evidence for the clinical meaningfulness of universal school-based MT.

The current study, set up and conducted before publication of the abovementioned findings, aimed to contribute to the current knowledge in three unique ways. The first unique contribution is the study's focus on both negative affect (NA) and positive affect (PA) related outcomes. That is to say, our study focuses on the potential impact of MT on emotional distress (i.e., depressive symptoms, anxiety and stress) and anhedonia (i.e., loss of pleasure or interest in previously enjoyable activities; APA, 2013). Emotional distress entails a mixture of affective responses, such as anxiety and depression, that all correspond with NA impairments (Matthews, 2016). Emotional distress (or subcomponents) is (are) commonly included as outcome variable(s) in school trials (see evidence above). However, as yet, no clear support has been provided for the beneficial impact of school-based MT on 10.13039/100006131PA in adolescents (e.g., Campbell, 2015; Sibinga, Webb, Ghazarian, & Ellen, 2016). On a correlational level, however, higher mindfulness in adolescents appears to be related to higher concurrent PA levels (Galla, 2016). Furthermore, in adults, a strong case has been made for the link between PA and mindfulness, as well as the impact MT may have on PA/anhedonia. For instance, mindfulness is positively associated with concurrent levels of PA (Jimenez, Niles, & Park, 2010; McLaughlin, Luberto, O’Bryan, Kraemer, & McLeish, 2019) and a higher state of mindfulness has been found predictive of higher PA in time series studies (Du, An, Ding, Zhang, & Xu, 2019). Via RCTs, MT has been shown to increase momentary PA (e.g., Geschwind, Peeters, Drukker, van Os, & Wichers, 2011; Lindsay et al., 2018) and decrease anhedonia (Cernasov et al., 2021).

A focus on anhedonia and ways to counteract this symptomatology is highly relevant in adolescence because it appears to be prevalent, although it often remains unrecognized (Gutkovich, 2014). Moreover, as a key symptom of depression, anhedonia in adolescents predicts a poor prognosis (i.e., longer and more severe depression course) and appears to be related to increased suicidal risk (Gutkovich, 2014). At a broader level, our approach aligns with an argument that to stepwise improve depression outcomes we need to attend to both PA and NA (Craske, Meuret, Ritz, Treanor, & Dour, 2016; Craske et al., 2019; Dunn, 2012).

The second unique contribution of this study involves the inclusion of three potential working mechanisms of MT on emotional distress and anhedonia (two at an intra-personal and one an inter-personal level). This follows the recommendation of a recent scoping review that future research in this field should focus on process evaluation, identifying key mechanisms that MT might target (Tudor et al., 2022). At an intra-personal level, vulnerable adolescents typically do not accept negative emotions, which paradoxically further increases them (e.g., Gross & Cassidy, 2019; Shallcross, Troy, Boland, & Mauss, 2010). They also tend to dampen positive emotions (e.g., Nelis, Luyckx, et al., 2016). Dampening is a response style towards positive emotions that reduces their intensity and duration through thoughts such as, “I do not deserve this positive feeling” or “This positive feeling will end soon” (Felver et al., 2016). Across age, dampening has been found to be positively related to depressive symptoms and anhedonia, concurrently and prospectively (Bean, Summers, & Ciesla, 2022). MT has the potential to unlock this challenging combination of response styles towards negative and positive emotions. Particularly, it is presumed that MT exerts its effect on emotional distress (related to NA) via lowering the tendency to not accept and/or suppress negative emotions. The (potential) positive impact on anhedonia (related to PA) is thought to be explained by a decrease in dampening of positive emotions. That is, during MT adolescents gradually learn to recognize and experience emotions and adopt a non-judgmental attitude. In that way, automatic habitual reactions towards emotions like non-acceptance/suppression of negative emotions and dampening of positive emotions, are expected to be replaced by alternative responses reflecting an open, non-judgmental attitude.

MT might also exert an effect at an inter-personal level. In adults, social norms focusing on pursuing happiness and dismissing negative emotions are likely to have unfavourable implications for wellbeing. For instance, social norms reflecting disapproval of negative emotions were found to increase NA in adult non-clinical samples (Bastian et al., 2012). Moreover, perceived social pressure not to experience/express negative emotions appeared to be predictive of depressive symptoms in adults in daily life (Dejonckheere, Bastian, Fried, Murphy, & Kuppens, 2017). Given the heightened importance of peer relationships during adolescence, sensitivity to social evaluation, and susceptibility to peer influence (Somerville, 2013; Steinberg & Monahan, 2007), similar social norms among peers might have deleterious effects in vulnerable adolescents. However, MT may act as an antidote to a social climate among peers characterised by social pressure not to experience and/or express negative emotions. Several mechanisms of change may be involved in turning the social climate towards one more conducive to flourishing. First, the open, non-judgmental attitude adolescents taught in the MT may transcend the individual level and help foster a classroom climate that promotes a more balanced recognition and acceptance of emotions, which is likely beneficial for adolescents’ well-being (Dejonckheere et al., 2017). Second, in-class MT may benefit peer relationships and peer acceptance through increases in prosocial behaviour (Andreu & García-Rubio, 2019; Donald et al., 2019; Schindler & Friese, 2022). Third, promising results have been found for the effectiveness of mindfulness-based interventions to increase empathy and compassion (Cheang, Gillions, & Sparkes, 2019). Finally, group connection and communication are facilitated and supported by a trainer who promotes a safe climate (e.g., ground rule of respect towards yourself and others; Cormack, Jones, & Maltby, 2018). This may have a normalising function (e.g., universality of suffering; Cormack et al., 2018), and eventually provide a platform to experience and express the full array of emotions. These mechanisms may all contribute to a healthier social climate characterised by less social pressure not to experience and express negative emotions.

The third unique contribution of this study is the use of Experience Sampling Methodology (ESM). As far as we are aware of, this is the first study in this field of research to rely on ESM. ESM is a validated, structured momentary assessment method that provides repeated, in-the-moment micro-measurements (i.e., in daily environment) of core psychological and behavioural variables in a prospective and ecologically valid manner (Csikszentmihalyi, 2014). Another strength of relying on this methodology is the reduction of recall bias related to retrospective measurements and consequently its higher reliability (Morris, 1999). Moreover, in mindfulness research ESM has been shown to be more sensitive to change compared to paper-and-pencil measures (e.g., Lindsay, Young, Brown, Smyth, & Creswell, 2019; Moore, Depp, Wetherell, & Lenze, 2016). Given the pioneering ESM approach in the field of universal school-based mindfulness research, self-report questionnaires (SRQs) were administered to corroborate the ESM results. In particular, any effects of MT were expected to be reflected in both ESM and SRQs data, irrespective of inherent unique features and strengths of both methodologies (e.g., in-moment vs. retrospective time perspective, higher external vs. internal validity, respectively).

In sum, following the PICO framework (Richardson, Wilson, Nishikawa, & Hayward, 1995), the overall aim of this trial was to evaluate the effects of a universal school-based MT (*Intervention*) for adolescents (*Population*) and to reveal underlying mechanisms using ESM. To this end, a cluster randomised controlled trial (MT vs. passive control group; *Comparison*) was conducted in secondary schools. In particular, concerning the effectiveness, MT was expected to lower (H1) anhedonia, and (H2) emotional distress (i.e., symptoms of depression, anxiety and stress; *Outcomes*). As regards the potential underlying mechanisms, MT was expected to exerts its effect via reductions in (H1) dampening of positive emotions, (H2) suppression/acceptance of negative emotions, and (H3) perceived social pressure towards the (non-)expression/(non-)experience of negative emotions.

## 1 Method

### 1.1 Selection of participants

After ethical approval (Ethics Committee Research UZ/KU Leuven, s62523) and preregistration of the study (ClinicalTrials.gov ID NCT04159272), participant recruitment started with contacting secondary schools in Flanders, Belgium (second and third grade of general education). Next, participating schools were invited to select a pair of parallel classes of adolescents (Dutch-speaking) for study enrolment. The study was open to all adolescents and no exclusion criteria were applied at individual or group level, except for a minimal group size of 12 adolescents (due to practical and methodological reasons). Adolescents and their parents received details about the study via information leaflets including the informed consent forms. Additionally, in-class information sessions were organised in which all study phases were explained. In total, five rounds of data collection were run between August 2019 and June 2, 022.^1^ In total, 13 pairs of classes enrolled in the study (recruited via 11 schools) with on average two to three schools in each round of data collection.

The final sample consisted of 231 participants, with 95 adolescents in the MT group (*M*_age_ = 15.7, *SD*_age_ = 0.97, range 14–18 years; gender: 70.53% females and 29.47% males) and 136 adolescents in the control group (*M*_age_ = 15.8, *SD*_age_ = 0.96, range 14–18 years; gender: 69.12% females, 29.41% males, 0.74% other and 0.74% no indication of gender). For both groups, the majority of participants indicated to be of Belgian ethnicity (79.41% and 80% respectively). Respectively, 11.58% and 11.03% of the participants mentioned one or two other ethnicities in addition to their Belgian ethnicity, and 8.42% and 11.03% mentioned to identify with another than the Belgian ethnicity (e.g., Turkish, Moroccan, French, Italian, Chinese, Romanian).

### 1.2 Study design and randomisation

The study employed a cluster randomised controlled design. After informed consent approval (by adolescents and parents) and baseline assessment, classes were randomly assigned to the intervention (i.e., MT) or passive control condition (i.e., regular curriculum). The randomisation process was conducted by an independent statistician using a computerised random number generator. All participants were assessed at three points in time, in a fixed order: before randomisation (T1; baseline or pre-intervention), post-intervention (T2; immediately after the intervention), and approximately three months after intervention (T3; follow-up^2^). Parallel classes completed the assessments at the exact same time points. The intended sample size of approximately 100 adolescents in both study arms (i.e., 200 in total), which was based on our experience with ESM data to reveal mechanisms of action (e.g., Van der Gucht, Dejonckheere et al., 2019), was reached.

### 1.3 Description of intervention

Adolescents in the MT condition were offered a MT programme at school during school hours. The MT was delivered by certified mindfulness trainers with extensive experience in working with adolescents, and a health-care education and/or background. Additionally, in each round of data collection, trainers received two group supervision sessions (90–120 min; halfway through and towards the end of the MT) organised by professional supervisors. Finally, regular (informal) meetings between the involved mindfulness trainers were organised. Given the design of the study, adverse events were not routinely being collected. However, a trainer becoming aware of an emerging safety issue would report this by sending a formal adverse event form to the principal investigator (FR).

The MT consisted of 8 sessions (90–100 min, 1 session/week for 8 consecutive weeks). Each session consisted of guided experiential mindfulness exercises (e.g., focus on the breath, body scan, breathing space, mindful yoga, insight meditation, walk meditation), sharing of experiences of these exercises, reflections in small groups, psycho-education (e.g., stress, depression, self-care), and review of home practices. The MT programme adheres to a standardized protocol developed from MBSR (Kabat-Zinn, 1990) and MBCT (Segal et al., 2002) manuals and was adjusted to an adolescent population.^3^ Adjustments were based on ample experience of mindfulness trainers (affiliated with our lab) with mindfulness and adolescents in different contexts (schools, clinical settings, refugee populations). The MT programme has been made available in an open source platform.^4^ Before this study, the MT programme was piloted and reviewed in an expert group of mindfulness trainers (supervised by dr. Adel Maex) and scientists working with youth in mental health care (Van der Gucht, Takano, Labarque et al., 2017) and in refugee centres (Van der Gucht, Glas, De Haene, Kuppens, & Raes, 2019). The in-class MT programme was supplemented with homework assignments and a mindfulness for adolescents smartphone application (You-Mind app, with audio material) to support further practice at home. The trainers encouraged adolescents to practise their skills on a daily basis for approximately 15 min. Adolescents in the control group received access to the mindfulness modules of the You-Mind app at the end of the study.

### 1.4 Procedure

This paper reports on the primary endpoints and related outcome measures.^5^ Data collection primarily relied on ESM, corroborated by SRQs. Analyses were conducted on both types of data to rule out the possibility that findings virtually rely on methodological artefacts. Despite the fact that ESM and SRQs tap into similar constructs in a slightly different way (i.e., in-moment vs. retrospective registration over a longer period of time), major effects were expected to be similarly reflected in ESM and SRQs data.

***Experience Sampling Methodology.*** In each assessment phase, the You-Mind app,^6^ installed on participants’ smartphone, beeped 10 times/day for 4 consecutive days. The timing of the beeps depended on a semi-stratified interval scheme (i.e., waking hours between 9 a.m. and 9 p.m. were divided into 10 equal intervals and in each interval 1 beep was randomly programmed). Each beep involved a series of 15 short questions (see *Measures*) that could be completed until 10 min after the beep was sent. In total, this ESM procedure yielded three bursts of time series data for each participant. Besides the administration and registration of ESM-questions and -responses, the You-Mind app also logged usage data. This included app starting times, visited audio and videos (including duration of the interaction), and app error logging. Registration ended when data collection was complete.

The minimal required compliance was initially set at 30%, as commonly used in ESM research (e.g., Kramer et al., 2014; van Winkel et al., 2017). However, during the course of data collection, many adolescents’ compliance fell below this cut-off. Consequently, the minimal required compliance for inclusion in data analysis was lowered to 20%. To further encourage participants to reach sufficiently high compliance levels, two changes to the initial study protocol were implemented from the fourth round of data collection onwards. First, compensation was made partially conditional on ESM compliance rates. Initially, participants received a voucher of a local, online store (Bol.com) of €10 for taking part in the study irrespective of adherence rates. However, in order to motivate participants to persistently engage in completing the ESM questions, participants in the two final data collection rounds were compensated with a voucher of €5 independent of adherence rates, and an additional one if they completed at least 75% of the ESM questions per assessment (i.e., 30 out of 40 beeps).

Second, general and individual email reminders were sent to participants during ESM assessment days. By sending general reminders (class-level), we aimed to keep participants motivated, remind them of the importance of ESM questionnaire completion, and prompt them to contact the study team if technical issues arose. In addition, participants with daily ESM compliance rates below 75% received an individual email with feedback on their compliance rate (e.g., Glenn, Nobles, Barnes, & Teachman, 2020) and they were encouraged to make an extra effort to increase their compliance rate(s) the next day(s).

***Self-Report Questionnaires.*** For the vast majority of the assessment points, a researcher visited the classes in person for the administration of the pen-and-paper SRQs during school hours. Completion time was approximately 45 min.

### 1.5 Measures

***Experience Sampling Methodology.*** Participants were presented 15 short questions per beep via the You-Mind app, of which 10 questions were relevant for the primary endpoints reported in this paper. Table 1 gives an overview of these questions and related outcome(s). Each question was presented with a Likert scale ranging from 0 (*not at all*) to 100 (*a lot or to a great extent*).

**Table 1:** ESM questions (primary endpoints).

Outcome	ESM Question
Emotional distress (3 items)	1. How anxious do you feel now? 2. How stressed do you feel now? 3. How depressed do you feel now?
Anhedonia (3 items)	1. To what extent do you now experience difficulties to enjoy activities? 2. To what extent do you expect to experience pleasure in the near future? *(reversed scored)* 3. To what extent do you feel happy now? *(reversed scored)*
(Non-)acceptance of Negative Emotions and Suppression of Negative Emotions (2 items)	1. Since the previous beep, to what extent were you able to accept negative feelings and let them be? *(reverse scored)* 2. Since the previous beep, to what extent did you try to suppress negative feelings?
Dampening (1 item)	Since the previous beep, to what extent did you think that positive feelings wouldn't last?
Expectations not to Experience Negative Emotions (1 item)	Since the previous beep, how much pressure did you feel from your peers not to feel anxious or depressed?

***Self-Report Questionnaires.*** Participants first completed questions about sociodemographic data (i.e., month and year of birth, age, gender, ethnicity, school, school grade, and school track), and continued with a series of SRQs (Dutch versions).

Symptoms of emotional distress were measured by the *Depression Anxiety Stress Scales* (DASS-21, 21 items; Lovibond et al., 1995). This scale is composed of three seven-item subscales, that assess the extent of depressive symptoms, anxiety and stress the participant experienced over the past week. Participants indicate the applicability of each symptom via a 4-point scale ranging from 0 (*not at all* or *never*) to 3 (*very much* or *most of the time*). A sample item for each of the subscales are respectively ‘I felt down-hearted and blue’, ‘I felt I was close to panic’, and ‘I found it difficult to relax’. For the three assessment points, good internal consistencies were found for the depression (α_T1_ = 0.840; α_T2_ = 0.870; α_T3_ = 0.863) and stress subscales (α_T1_ = 0.805; α_T2_ = 0.851; α_T3_ = 0.879), and acceptable to good internal consistencies were found for the anxiety subscale (α_T1_ = 0.741; α_T2_ = 0.793; α_T3_ = 0.838).

The *Leuven Anhedonia Self-report Scale* (2nd version; LASS; Nelis, Bastin, Raes, & Bijttebier, 2018) was used to assess anhedonia. This scale consists of 12 items that tap into three aspects of anhedonia, namely consummatory (i.e., reduced pleasure in ongoing experiences), anticipatory (i.e., diminished pleasure from anticipation to a future positive event), and motivational (i.e., decreased drive or motivation to pursue positive outcomes or reward). The applicability of statements for the last two weeks was evaluated on a 5-point scale ranging from *completely false* (1) to *completely true* (5), with a higher total score reflecting higher levels of anhedonia. Sample items are ‘I found little pleasure in things that I used to enjoy’ and ‘I was not motivated to do all kinds of things’. Excellent internal consistencies were found for the three time points in the current sample (α_T1_ = 0.921; α_T2_ = 0.940; α_T3_ = 0.944).

Dampening responses to positive affect were measured via the dampening subscale of the *Responses to Positive Affect* scale (RPA; Feldman, Joormann, & Johnson, 2008). Higher scores indicate a higher tendency to engage in dampening. Participants rated the applicability of the items on a Likert scale, ranging from 1 (*almost never*) to 4 (*almost always*). In this study, only six items of the original English 8-item scale were presented. One item of the original English version of the scale was not retained in the Dutch version (Raes et al., 2009), and another item appeared to load remarkably low on the dampening factor in adolescents (Nelis, Luyckx, et al., 2016). Two examples are ‘When you feel happy, how often do you think “I do not deserve this”?’ or ‘When you feel happy, how often do you think about things that could go wrong?‘. Total scores for the dampening subscale of the RPA showed acceptable to good internal consistencies for the three assessment points (α_T1_ = 0.779; α_T2_ = 0.827; α_T3_ = 0.847).

The 10-item *Non-Acceptance and Suppression of Negative Emotions* Scale (Raes, 2019; unpublished; Table A1; Appendix A) makes use of a 7-point scale (1 refers to *not at all applicable*, 7 refers *very much applicable*) to examine the extent to which individuals usually respond towards negative emotions with non-acceptance and suppression. In other words, higher scores indicate higher levels of non-acceptance and suppression of negative emotions. Two sample items are, respectively, ‘I find it difficult to accept negative feelings’ and ‘I try to suppress negative feelings’. For both the non-acceptance and suppression subscale of the NASNES, good to excellent internal consistencies were found for the three assessment points (respectively, α_T1_ = 0.886; α_T2_ = 0.900; α_T3_ = 0.912 and α_T1_ = 0.845; α_T2_ = 0.884; α_T3_ = 0.912).

Social expectancies towards the (non-)expression of negative thoughts and emotions were evaluated via the adapted and extended 26-item *Social Expectancies to experience Depression and Anxiety Scale* (SEDAS; Bastian et al., in preparation; McGuirk, Kuppens, Kingston, & Bastian, 2018; Table A2, Appendix A). For this study, the SEDAS was adapted to the class social climate and, for exploratory reasons and beyond the scope of this article, extended to social expectancies about the (non-)experience/expression of positive thoughts and emotions. Participants rated the extent to which they agreed with particular statements on a Likert scale ranging from 1 (*totally disagree*) to 9 (*totally agree*). For instance, ‘I think my classmates accept individuals who feel depressed of anxious and consider them as normal’ reflects the absence of perceived pressure not to feel/express negative emotions. Total scores for the subscales about negative thoughts/emotions showed good to excellent internal consistency in this data set at the three assessment points (α_T1_ = 0.851, α_T2_ = 0.861, α_T3_ = 0.866).

Participants from the MT group completed additional questions at post-intervention and follow-up. At post-intervention, participants reported on the frequency of home practice during the training, both for formal (i.e., practice with the type of exercises offered during the MT) and informal practice (i.e., in daily life). At follow-up, similar questions were used to learn more about the frequency of home practice during the follow-up period. At this final assessment point, adolescents also rated the degree of helpfulness of the app to practise mindfulness skills at home, their frequency of use, and the quality of the app (i.e., user friendliness). Finally, three self-experienced positive and negative aspects/effects of the MT were questioned (‘What did you experience as pleasurable or positive vs. pleasurable or difficult? What did you experience as positive vs. negative effects? Below you can mention up to three positive and negative aspects.‘).

### 1.6 Data analyses

Preliminary analyses, including drop-out analyses and analyses to detect baseline imbalance, were conducted to examine sample characteristics. Afterwards, as preregistered and similar to the approach adopted in Van der Gucht, Takano, Kuppens, and Raes (2017) and Van der Gucht, Takano, Raes, and Kuppens (2018), hypotheses were examined with intention-to-treat analyses. General linear modelling and multilevel mixed effects modelling formed the basis for these analyses. In particular, to test the intervention effect on outcomes (anhedonia H1 and emotional distress H2), and putative mediators (dampening H1, acceptance/suppression of negative feelings H2, perceived social experiences not to experience negative emotions H3), piecewise multilevel models with three levels were used. Time points (Level-1) were nested within persons, and persons (Level-2) were nested within schools (Level-3). In this model, (a) the dummy-coded assessment time (as Level 1-variabele; two dummy-coded variables T2 and T3), (b) the treatment condition (as a Level-2 variable), and (c) their cross-level interactions were included to predict the outcome. The significance level (α = 0.05) was corrected for multiple comparisons according to the method described by Benjamini and Hochberg (1995). The model was specified as follows: $Y_{ijk}=\beta_{0jk}+\beta_{1jk}*T2+\beta_{2jk}*T3+\beta_{3}*{CV}_{j}+r_{ijk}$

$Y_{ijk}$ represents the outcome on the i*th* time point of the *j*th participant of the *k*th school. The right half of the equation consists of the Level-1 intercept ($\beta_{0jk}$), the slopes for T2 ($\beta_{1jk}$), T3 ($\beta_{2jk}$) and the covariates (age mean centered and gender, CV; $\beta_{3}$), of which the intercept and slopes for T2 and T3 allowed to vary randomly across persons at Level-2 and schools at Level-3. The final term in the equation is $r_{ijk}$, which represents the residual.

The random intercepts ($\beta_{0jk}$) and slopes $(\beta_{1jk}$; $\beta_{2jk}$) varied across persons and schools (Level-2 and Level-3), and their associations with condition (dummy coded: ‘1’ for the intervention and ‘0’ for the control group) were coded as follows:

$$\beta_{0jk}=\beta_{00k}+\beta_{01k}\;x{Condition}_{jk}+u_{0jk}$$

$$\beta_{1jk}=\beta_{10k}+\beta_{11k}\;x{Condition}_{jk}+u_{1jk}$$

$$\beta_{2jk}=\beta_{20k}+\beta_{21k}x{Condition}_{jk}+u_{2jk}$$

Individual differences that cannot be explained by condition differences were modelled as the random effects ($u_{0jk},u_{1jk}$, and $u_{2jk})$. The intercept ($\beta_{00k})$, main effects ($\beta_{01k}$, $\beta_{10k}$, and $\beta_{20k}$) and cross-level interactions between assessment time and condition ($\beta_{11k}$, $\beta_{21k}$) were allowed to vary across schools.

After the outcome analyses (i.e., treating outcomes and putative mediators as dependent variable), mediation analyses were planned via the estimation of a time-lagged mediation model for the MT group. Central to these planned analyses was that the proposed mediators served as lagged time-varying predictors (i.e., change in mediator from T1 to T2) of subsequent changes in the outcome. This approach was based on the procedure outlined by Bauer, Preacher, and Gil (2006). Finally, re-estimation of a model on the whole sample (including MT and control group) was planned to test if the mediation effect was moderated by condition. However, in the light of the results of the outcome analyses (see *Results*), the planned analyses on the mediation effects were not conducted.

For both the ESM and SRQ data, hierarchical linear modelling was conducted using the “lme 4” package (R software, version 4.2.0). For each outcome, a top-down process of model comparison was conducted, which resulted in models that were slightly different in terms of the random effect parameters included. In particular, following the approach of Barr, Levy, Scheepers, and Tily (2013), convergence and singularity problems (i.e., overfitted models) were resolved by systematically removing random effect parameters for which the variances were estimated as zero, or for which correlations were estimated as close to an absolute value of 1.

## 2 Results

### 2.1 Preliminary analyses

**Fig. 1:**
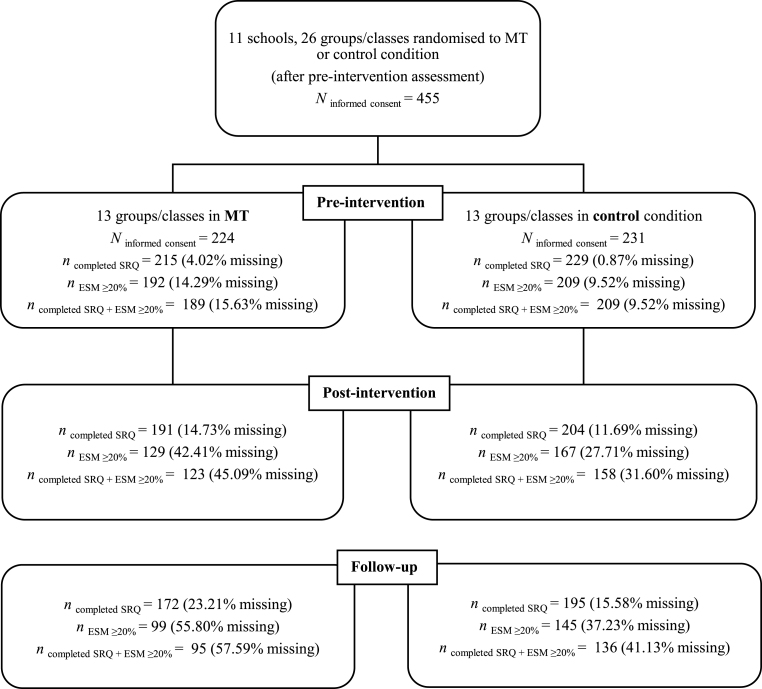
Flowchart of the Recruitment and the Retention of Participants in the Trial. *Note.* Percentages of missing values refer to the number of participants with no or too little data (e.g., too low ESM compliance) divided by those who provided informed consent.

**Participant Flow.** The participant flow^7^ is presented in Fig. 1. For the SRQs, missing values mainly resulted from absence from school on the day of testing (e.g., medical reasons). A minority of participants dropped out because of a switch to another class/school during the course of the study and a small number of participants from the intervention group (*n*_total_ = 10) formally withdrew from the study (without officially sharing a reason). During the entire course of the trial, mindfulness trainers did not report any adverse events or emerging safety issues. Given the reliance on SRQ to corroborate the ESM results, participants of whom SRQ data were available (after mean imputation if one or two missing values were observed at item-level per scale) and a minimum ESM compliance of 20% for the three assessment points were retained for the main analyses (see *Participants*).

**Descriptive Statistics.**Table 2 presents the descriptive statistics of all outcomes for both groups at each assessment point. Standardized mean differences (SMD) were calculated to detect baseline imbalance (Schober & Vetter, 2019). As indicated, the conventional cut-off of an absolute SMD of 0.10 is slightly exceeded for some outcomes, which indicates mild baseline imbalance between both groups. For depressive symptoms (measured with DASS-D; Lovibond & Lovibond, 1995) considerable baseline imbalance is observed (*SMD* = 0.251) with a significantly higher mean level of depressive symptoms reported for the intervention group compared to the control group.

**Table 2:** Descriptive statistics for raw outcome scores at each assessment point.

Variable	T1 *M (SD)*		*SMD*	T2 *M (SD)*		T3 *M (SD)*	
	Control	MT		Control	MT	Control	MT
**ESM measures**							
Depressed	25.5 (29.0)	27.2 (29.4)	.058	21.6 (25.2)	21.4 (25.4)	26.4 (27.5)	25.8 (27.0)
Anxious	18.1 (23.6)	20.5 (25.0)	.102*	22.2 (24.5)	24.3 (26.7)	23.0 (24.6)	23.8 (25.1)
Stressed	28.6 (27.5)	30.3 (29.3)	.062	31.1 (28.8)	34.3 (30.2)	27.5 (27.0)	28.6 (27.3)
Anhedonia	37.0 (19.7)	37.8 (20.5)	.039	37.0 (21.3)	36.6 (21.6)	41.4 (19.4)	39.6 (19.0)
Non-acceptance NA	51.1 (29.8)	50.1 (29.9)	.035	47.1 (27.9)	46.6 (27.6)	56.8 (30.0)	53.1 (30.5)
Suppression NA	33.8 (28.2)	36.1 (27.8)	.081	28.6 (25.3)	31.2 (26.5)	23.4 (25.3)	24.1 (26.1)
Dampening PA	28.4 (27.8)	30.6 (28.0)	.080	24.2 (24.8)	25.7 (26.1)	33.8 (30.0)	32.9 (29.0)
Expectations not to experience NA	13.7 (21.8)	17.1 (24.2)	.147*	11.1 (18.7)	15.0 (23.4)	23.7 (29.7)	24.3 (28.5)
**SRQ measures**							
Depressive symptoms (DASS-D)	8.77 (8.31)	11.03 (9.68)	.251*	10.53 (9.52)	8.80 (8.99)	9.86 (8.15)	9.89 (10.41)
Anxious symptoms (DASS-A)	8.87 (7.07)	8.68 (8.15)	.024	11.03 (7.87)	10.41 (9.76)	10.72 (8.40)	9.89 (10.53)
Stress (DASS-S)	11.74 (8.35)	12.28 (8.15)	.065	13.00 (8.44)	14.21 (9.21)	12.84 (9.21)	13.52 (10.80)
Anhedonia (LASS)	22.77 (9.56)	24.15 (10.34)	.138	24.13 (10.59)	23.19 (9.69)	24.45 (10.40)	23.68 (11.07)
Non-Acceptance NA (NASNES-NA)	19.31 (7.13)	19.23 (7.33)	.010	18.20 (7.26)	17.45 (7.27)	18.38 (7.11)	16.87 (7.66)
Suppression NA (NASNES-S)	23.47 (6.36)	22.70 (6.98)	.116*	21.75 (7.13)	20.83 (7.11)	21.43 (7.19)	19.52 (8.59)
Dampening PA (RPA-D)	10.88 (3.75)	11.18 (4.10)	.077	10.51 (3.66)	10.49 (4.07)	13.38 (3.56)	10.43 (4.31)
Perceived social pressure non-experience/expression of NA (SEDAS-NEG)	56.89 (14.58)	55.11 (17.64)	.111*	52.95 (17.39)	50.62 (16.79)	51.65 (18.95)	47.51 (17.22)

*Note. N*_control_ = 136; *n*_intervention_ = 95; T1 = baseline, T2 = post-intervention, T3 = follow-up; NA = Negative Affect/emotions, PA = Positive Affect/emotions; SMD = Standardized Mean Difference, >.10 are indicated by an *.

Concerning the distribution in terms of depressive symptom severity (Appendix B), the majority of participants in the control and MT group (resp. 72.79% and 68.42%) reported mild or normal levels of depressive symptoms. However, still up to one third of the adolescents in the control (27.2%) and in the MT group (31.58%) reported moderate or (extremely) severe levels of depressive symptoms at baseline, leaving considerable room for symptom improvement. It should be noted though that a clear difference was observed at the higher end of the continuum. Here, a higher percentage of participants in the MT vs. control group reported (extremely) severe levels of depressive symptomatology (17.90% vs. 7.35%).

### 2.2 Multilevel analyses

***ESM data.***Table 3 gives an overview of the fixed effects’ estimations for the models for the main outcomes (anhedonia and emotional distress) based on the ESM data. Regarding anhedonia (H1), no significant fixed condition × time interaction effects were found at T2 or T3. For emotional distress (H2; depressive symptoms, anxiety, stress, no significant interaction effects were observed either. So, neither at post-intervention nor at follow-up, clear symptom reductions were observed for the MT (vs. control) group. Similar models were run for the putative mediators as outcomes (Table 4). Concerning dampening of positive emotions (H1), suppression/non-acceptance of negative emotions (H2), and perceived social pressure not to experience/express negative emotion (H3), no significant condition x time effects were observed.^8^

**Table 3:** Main and interaction fixed effects of multilevel model estimating main outcomes (H1 and H2; ESM data).

	Anhedonia (H1)						Emotional Distress (H2)					
	Difficulties to Enjoy		No Expected Pleasure		Not Feeling Happy		Depressive		Anxious		Stressed	
	Est. (SE)	*t* (*p) df*	Est. (SE)	*t* (*p) df*	Est. (SE)	*t* (*p) df*	Est. (SE)	*t* (*p) df*	Est. (SE)	*t* (*p) df*	Est. (SE)	*t* (*p) df*
Intercept	−0.20 (0.08)	−2.43 (.02)* 77.11	−0.20 (0.09)	−2.11 (0.04)* 35.22	−0.15 (0.16)	−0.94 (.36) 13.42	−0.14 (0.18)	−0.85 (.41) 14.57	−0.46 (0.09)	−5.15 (<.001)** 59.95	−0.45 (0.09)	−5.11 (<.001)** 34.77
Age	0.11 (0.04)	2.49 (.03)* 10.66	0.09 (0.06)	1.57 (0.13) 26.13	0.05 (0.06)	0.76 (.45) 205.77	0.08 (0.09)	1.07 (.29) 189.43	0.12 (0.05)	2.60 (.03)* 8.19	0.07 (0.05)	1.53 (0.18) 6.15
Gender	0.24 (0.08)	2.8 (.005)* 226.24	0.11 (0.09)	1.28 (0.20) 222.85	0.12 (0.07)	1.70 (.09) 217.49	0.22 (0.09)	2.41 (.02)* 217.24	0.41 (0.09)	4.33 (<.001)** 225.64	0.52 (0.09)	5.78 (<.001)** 225.72
T2	0.02 (0.04)	0.42 (.68) 225.09	0.20 (0.05)	4.41 (<.001)** 227.10	−0.15 (0.14)	−1.05 (.31) 11.68	−0.07 (0.15)	−0.49 (.64) 12.60	0.18 (0.05)	3.41 (<.001)** 224.81	0.12 (0.06)	1.90 (.08) 14.68
T3	0.04 (0.05)	0.76 (.45) 228.11	0.17 (0.05)	3.22 (0.001)* 229.39	0.15 (0.12)	1.28 (.22) 12.32	0.01 (0.13)	0.10 (.92) 13.49	0.21 (0.05)	3.89 (<.001)** 229.61	0.05 (0.08)	0.62 (.54) 14.74
Condition	0.06 (0.08)	0.71 (.48) 227.25	0.01 (0.08)	0.15 (0.88) 222.72	0.07 (0.08)	0.80 (.43) 217.69	0.09 (0.10)	0.91 (.37) 218.07	0.14 (0.09)	1.50 (0.14) 225.93	0.08 (0.09)	0.92 (.36) 226.69
T2:Condition	−0.03 (0.07)	−0.50 (.62) 225.29	−0.06 (0.07)	−0.85 (.40) 227.37	−0.02 (0.10)	−0.26 (.80) 221.01	−0.04 (0.10)	−0.43 (.67) 220.34	−0.028 (0.08)	−0.35 (0.73) 225.19	0.04 (0.07)	0.53 (.60) 225.01
T3:Condition	−0.11 (0.08)	−1.38 (.17) 228.63	−0.03 (0.08)	−0.42 (0.68) 229.92	−0.04 (0.09)	−0.47 (.64) 227.62	−0.06 (0.10)	−0.59 (.56) 224.79	−0.08 (0.08)	−0.99 (0.33) 230.15	0.003 (0.08)	0.05 (.96) 228.75

*Note*. *p < .05; **p < .001; Est. = Estimate, SE = Standard Error; *n*_control_ = 136; *n*_intervention_ = 95.

**Table 4:** Main and interaction fixed effects of multilevel model estimating outcomes putative mediators (H1, H2, and H3; ESM data).

	Dampening PA (H1)		Suppression NA (H2)		Non-Acceptance NA (H2)		Pressure no NA (H3)	
	Est. (SE)	*t* (*p) df*	Est. (SE)	*t* (*p) df*	Est. (SE)	*t* (*p) df*	Est. (SE)	*t* (*p) df*
Intercept	−0.13 (0.14)	−0.97 (.34) 19.68	0.04 (0.11)	0.37 (.71) 25.10	−0.10 (0.13)	−0.81 (.43) 20.18	−0.13 (0.12)	−1.06 (.30) 20.96
Age	0.09 (0.08)	1.14 (.26) 86.71	−0.02 (0.07)	−0.29 (.78) 16.06	−0.03 (0.07)	−0.40 (.70) 77.53	0.09 (0.07)	1.26 (.21) 86.05
Gender	0.18 (0.10)	1.84 (.07) 218.96	0.15 (0.10)	1.49 (.14) 221.08	0.13 (0.09)	1.46 (.15) 219.69	−0.007 (0.09)	−0.08 (.94) 218.85
T2	−0.14 (0.09)	−1.50 (.16) 12.67	−0.19 (0.07)	−2.75 (.01)* 16.83	−0.11 (0.10)	−1.17 (.27) 12.80	−0.09 (0.09)	−1.06 (.31) 14.87
T3	0.07 (0.12)	0.60 (.56) 12.86	−0.34 (0.11)	−3.18 (.005)* 15.54	0.12 (0.10)	1.21 (.24) 15.90	0.29 (0.14)	2.08 (.06) 11.75
Condition	0.10 (0.10)	1.00 (.32) 217.38	0.11 (0.10)	1.05 (.30) 221.10	−0.01 (0.10)	−0.08 (.94) 217.31	0.15 (0.09)	1.68 (.09) 220.48
T2:Condition	−0.03 (0.09)	−0.29 (.77) 221.38	0.02 (0.09)	0.22 (.83) 224.68	0.04 (0.10)	0.44 (.66) 221.65	0.02 (0.07)	0.28 (.78) 224.95
T3:Condition	−0.11 (0.09)	−1.23 (.22) 223.97	−0.01 (0.11)	−0.11 (.91) 225.87	−0.06 (0.10)	−0.61 (.54) 227.47	−0.10 (0.10)	−1.00 (.32) 223.76

*Note*. *p < .05; **p < .001; Est. = Estimate, SE = Standard Error; *n*_control_ = 136; *n*_intervention_ = 95.

***SRQ data.*** Similar hierarchical linear models were run for the outcomes measured via the SRQs. Table 5 gives an overview of the results for the fixed effect parameters for the main outcomes. For anhedonia (H1) and depressive symptoms (subcomponent of emotional distress; H2), significant condition x time effects were found at T2 (resp., Estimate = −0.22, *p* = .03; Estimate = −0.44, *p* = .02). However, these effects did not withstand correction for multiple testing (*p* = .12; Benjamini & Hochberg, 1995). No significant condition x time effects were found for the putative mediators treated as outcomes (Table 6).

**Table 5:** Main and interaction fixed effects of multilevel model estimating main outcomes (H1 and H2; SRQ data).

	Anhedonia (H1)		Emotional Distress (H2)					
			Depressive		Anxious		Stressed	
	Est. (SE)	*t* (*p) df*	Est. (SE)	*t* (*p) df*	Est. (SE)	*t* (*p) df*	Est. (SE)	*t* (*p) df*
Intercept	−0.26 (0.14)	−1.88 (.08) 16.72	−0.35 (0.10)	−3.51 (<.001)** 355.62	−0.50 (0.12)	−4.22 (<.001)** 268.89	−0.58 (0.11)	−5.13 (<.001)** 279.02
Age	0.07 (0.07)	0.91 (.39) 7.51	0.05 (0.04)	1.40 (0.16) 686.35	−0.04 (0.06)	−0.68 (.50) 215.76	0.06 (0.06)	1.01 (.32) 201.99
Gender	0.24 (0.12)	1.97 (.05) 219.32	0.33 (0.08)	4.02 (<.001)** 686.35	0.52 (0.12)	4.32 (<.001)** 221.47	0.62 (0.11)	5.42 (<.001)** 221.84
T2	0.13 (0.07)	1.92 (0.06) 257.86	0.19 (0.12)	1.62 (0.11) 457.35	0.25 (0.08)	3.08 (.005)* 24.73	0.15 (0.08)	1.82 (.07) 18.00
T3	0.17 (0.07)	2.37 (.02)* 354.32	0.12 (0.12)	1.02 (0.31) 457.70	0.22 (0.09)	2.56 (.02)* 18.00	0.13 (0.10)	1.36 (.19) 13.74
Condition	0.08 (0.19)	0.42 (.68) 14.02	0.26 (0.13)	2.04 (0.04)* 229.19	−0.01 (0.13)	−0.90 (.93) 319.94	0.09 (0.12)	0.72 (.47) 336.35
T2:Condition	−0.22 (0.11)	−2.12 (.03*/.12)⸷ 257.86	−0.44 (0.19)	−2.36 (0.02*/.12)⸷ 457.35	−0.05 (0.11)	−0.45 (.66) 287.09	0.08 (0.11)	0.66 (.51) 282.57
T3:Condition	−0.22 (0.11)	−1.92 (.06) 354.32	−0.25 (0.18)	−1.33 (0.18) 457.70	−0.08 (0.12)	−0.65 (.51) 344.57	−0.02 (0.17)	−0.09 (.93) 12.92

*Note*. *p < .05; **p < .001; Est. = Estimate, SE = Standard Error; *n*_control_ = 136; *n*_intervention_ = 95; Depressive symptoms, symptoms of anxiety and stress were measured via the respective subscales of the DASS-21 (Lovibond & Lovibond, 1995); ⸷ Effect no longer significant after Benjamini-Hochberg (BH) correction for multiple testing, respectively uncorrected and BH-corrected *p*-value between brackets.

**Table 6:** Main and interaction fixed effects of multilevel model estimating outcomes putative mediators (H1, H2, and H3; SRQ data).

	Dampening (H1)			Suppression NA (H2)		Non-Acceptance NA (H2)			Pressure non-expression/experience of NA (H3)	
	Est. (SE)	*t* (*p) df*		Est. (SE)	*t* (*p) df*	Est. (SE)	*t* (*p) df*		Est. (SE)	*t* (*p) df*
Intercept	−0.19 (0.13)		1.54 (.13) 265.43	0.14 (0.12)	1.22 (.22) 280.73	−0.16 (0.12)		−1.35 (.18) 294.59	0.28 (0.12)	2.32 (.02)* 260.66
Age	0.05 (0.06)		0.79 (.43) 225.00	−0.04 (0.06)	−0.76 (.45) 224.64	0.03 (0.06)		0.44 (.66) 224.99	0.09 (0.06)	1.47 (.14) 231.97
Gender	0.36 (0.13)		2.78 (.006)* 225.00	0.14 (0.12)	1.17 (.25) 224.64	0.42 (0.12)		3.55 (.005)* 224.99	−0.06 (0.13)	−0.47 (.64) 221.77
T2	−0.11 (0.06)		−1.71 (.09) 454.00	−0.23 (0.07)	−3.18 (.002)* 264.38	−0.16 (0.08)		−2.10 (.04)* 260.98	−0.26 (0.08)	−3.31 (.004)* 15.92
T3	−0.13 (0.06)		−2.16 (.03)* 454.00	−0.29 (0.08)	−3.49 (<.001)** 346.79	−0.14 (0.08)		−1.73 (.08) 379.52	−0.34 (0.09)	−3.96 (.001)* 16.16
Condition	0.08 (0.13)		0.59 (.56) 322.23	−0.10 (0.13)	−0.81 (.42) 342.31	−0.01 (0.13)		−0.05 (.96) 363.70	−0.10 (0.13)	−0.75 (.45) 305.31
T2:Condition	−0.07 (0.10)		−0.75 (.46) 454.00	−0.02 (0.11)	−0.22 (.83) 264.38	−0.08 (0.12)		−0.68 (.50) 260.98	0.01 (0.10)	0.09 (.93) 261.71
T3:Condition	−0.06 (0.10)		−0.62 (.53) 454.00	−0.15 (0.13)	−1.20 (.23) 346.79	−0.19 (0.12)		−1.52 (0.13) 379.52	−0.12 (0.11)	−1.10 (.27) 347.31

*Note*. *p < .05; **p < .001; *n*_control_ = 136; *n*_intervention_ = 95.

All abovementioned analyses were rerun using a wild bootstrap procedure (“lmeresampler” R package, Loy & Korobova, 2021) as distributional assumptions on the error terms nor homoscedasticity are required (Modugno & Giannerini, 2015). Conclusions based on the 95% confidence intervals (CIs, uncorrected) remained largely identical (Appendix C: Table C1, C2; Appendix D: Table D1, D2). To rule out potential confounding or moderating factors (i.e., exclusion of participants based on ESM compliance, mean level of ESM compliance, baseline depressive symptoms, frequency of home practice), post-hoc exploratory analyses were conducted. Generally, findings and conclusions remained unchanged (Supplementary Material for details).

### 2.3 Engagement with MT

Self-reported levels of frequency of home practice (Table 7) are considered to reflect the level of actual engagement with the MT. Overall, only a minority of participants reported actively engaging with the home practice. In particular, for formal and informal practice respectively, up to 21% and 32% of the participants indicated to have practised at least once a week during the course of the MT. After the MT was finished, these percentages dropped to 8% and 26%. Concerning the type of practice, both during the MT at school and during the follow-up period, reported levels of informal practice slightly exceeded levels of formal practice. Especially for formal practice, self-reported levels of home practice during the course of the MT appeared to be somewhat higher compared to the follow-up period.

**Table 7:** Percentages and numbers of participants of the intervention group subdivided into categories based on self-reported frequency of home practice (informal and formal) during the intervention (MT) and during the follow-up.

Frequency of Home Practice (categorical)	During the Intervention (MT) (reported at T2)		During the Follow-Up Period (reported at T3)	
	Formal % (*n*)	Informal % (*n*)	Formal % (*n*)	Informal % (*n*)
Never (0)	34.07 (31)	29.21 (26)	45.26 (43)	36.84 (35)
Once (1)	23.08 (21)	16.85 (15)	20.00 (19)	13.68 (13)
Once/month (2)	7.69 (7)	8.99 (8)	18.95 (18)	12.63 (12)
Several times/month, but not weekly (3)	14.29 (13)	13.48 (12)	7.37 (7)	11.58 (11)
Once/week (4)	9.89 (9)	10.11 (9)	4.21 (4)	10.53 (10)
Several times/week, but not daily (5)	8.79 (8)	13.48 (12)	1.05 (1)	11.58 (11)
Once/day (6)	2.20 (2)	4.49 (4)	3.16 (3)	2.11 (2)
Several times/day (7)	0 (0)	3.37 (2)	0 (0)	1.05 (1)

Participants also indicated the average duration of formal home practice during the course of the MT and/or follow-up period (Table 8). During the MT, about 50% of the participants who practised by means of formal exercises chose the option ‘≤ 5 min’. About one third reported to have used exercises of on average 10 min. Smaller groups reported engaging in exercises of about 15 and 20 min on average (resp., 14.29% and 4.76%). During the follow-up period, again a considerable group of participants reported engaging with formal exercises taking ≤5 min on average (68.97%). About 17% of the participants used exercises of around 10 min on average, and smaller percentages of participants formally practised for 15 or 20 min on average (resp., 10.34% and 3.15%).

**Table 8:** Self-reported average duration of home practice for formal and informal practice (reported at T2 and T3).

Average Duration of Home Practice	During the Intervention (MT) (reported at T2)	During the Follow-Up Period (reported at T3)
Formal (categorical)	*n* = 63	*n* = 58
Less than 5 min	21 (33.33%)	24 (41.38%)
5 min	11 (17.46%)	16 (27.59%)
10 min	19 (30.16%)	10 (17.24%)
15 min	9 (14.29%)	6 (10.34%)
20 min	3 (4.76%)	2 (3.45%)
30 min	0	0
Longer than 30 min	0	0
Informal (open question; in minutes)	*M* = 7.856	*M* = 6.321
	*SD* = 7.699	*SD* = 7.681
	Min = 1, Max = 35	Min = 1, Max = 40

*Note.* The open question to assess the average duration of informal practices was formulated as follows: “When doing an informal mindfulness exercise, how many minutes did you devote to it on average?”

Regarding informal practice (e.g., paying attention to environment on way to school), participants reported the average duration of their practice. During the course of the MT and the follow-up, participants estimated to have practised or applied mindfulness skills in an informal way for respectively 7.86 (*SD* = 7.70) and 6.32 (*SD* = 7.69) minutes. App loggings on the duration of interaction with audio files on the You-Mind app, another measure of MT engagement, are added in the Supplementary Material.

### 2.4 Self-experienced positive and negative aspects related to the MT

As part of the SRQs participants provided us with positive and negative experiences related to the MT. The broad variety of answers reflects considerable differences in adolescents' attitudes towards and experiences of the MT. The three positive aspects most frequently mentioned were that the MT (a) induced a sense of tranquillity or relaxation (*n* = 118; e.g., ‘It was relaxing’, ‘It helped me to calm down’), (b) reduced feelings of stress or changed the way of responding towards negative emotions and stress in particular (*n* = 42; e.g., ‘less stress’, ‘learn how to deal with difficult feelings’), and (c) helped with attention and awareness (*n* = 91; e.g., ‘improved attention’, ‘becoming more aware of my body and environment’). The most frequently mentioned negative experiences were (a) negative attitude towards mindfulness (*n* = 67; e.g., ‘no need’, ‘boring’, ‘maybe a bit too young to think about yourself that much’, ‘a bit silly and boring’). The other two most frequently mentioned aspects were of a practical nature, namely referring to (b) the time investment (*n* = 49; e.g., ‘It takes (too much) time’, ‘I prefer to invest my time in other things’, ‘It is a loss of time’), and (c) the duration of sessions and exercises (*n* = 37; e.g., ‘some exercises were too long’, ‘a bit long-winded’, ‘duration of sessions was too long’). A more extensive overview of participants' answers is added to the Supplementary Material.

## 3 Conclusion and discussion

Concerning our main outcomes, no evidence was found for a significant impact of MT on levels of anhedonia and levels of emotional distress. In addition, there was no evidence that the MT exerts an effect on any of the hypothesized underlying mechanisms (dampening, non-acceptance/suppression of NA, and perceived social pressure to non-experience/express NA or experience/express PA). The exploratory analyses did not yield support for the moderating role of level of ESM compliance or baseline depressive symptomatology. Conclusions did not change in any notable way when applying bootstrapping or when taking into account all available data irrespective of ESM compliance.

The SRQs did reveal a tendency in the MT group towards lower levels of depressive symptoms and, to a certain extent, towards lower levels of anhedonia (i.e., no longer when taking into account all SRQ data available) at post-intervention. However, it should be mentioned that SRQs were included to corroborate ESM findings and sample size planning was based on the ESM data structure. Methodological artefacts may explain the differences between the findings for the ESM and SRQ data. Participants' different time perspectives in the two methodologies may be the first explanatory factor. That is, for the ESM assessments, adolescents reported on their in-moment feelings/experiences (e.g., ‘How stressed do you feel *now*?‘) or took a short-term retrospective view on their emotion regulation strategy use (e.g., ‘*Since the previous beep*, to what extent did you suppress negative emotions?‘). For the SRQs, the considered time perspective was considerably longer (i.e., past one or two weeks), increasing the risk for self-report biases. A second explanatory factor may be related to the number of items used to measure the outcomes. For instance, for depressive symptoms one single ESM question (i.e., ‘How depressed do you feel now?‘) was used, while the SRQ relied on multiple items tapping into different facets. This rather narrow focus in the ES assessments may have restricted the range of variance and therefore the opportunity to observe differences over time. Alternatively, it might be that MT more strongly impacts those symptoms not assessed via ESM (e.g., attenuation negative self-view).

Nevertheless, irrespective of the observed (short-term) trend based on the SRQ data, the current study did not reveal substantial and/or long-lasting significant effects. Therefore, overall, our findings align with the conclusion of recently published reviews in the field. Summarised effect sizes suggested that (universal) school-based MTs only have the power to yield small effects, that often become insignificant in comparison with an active control group, which may call into question their (unique) clinical meaningfulness (Fulambarkar et al., 2022; Phillips & Mychailyszyn, 2022). In addition, our conclusion generally dovetails with the recently published findings of the MYRIAD trial. This fully powered, rigorous cluster RCT did not find support for the superiority of universal school-based MT compared to normal provision of social-emotional education in early adolescents (Kuyken et al., 2022). Notably, our trial is not directly comparable with the MYRIAD trial due to some major differences. First, our trial focused on mid-adolescents while the MYRIAD trial sample was composed of early adolescents (11–14 years). Second, the MT delivered in the MYRIAD trial (i.e., .*b Mindfulness in Schools* curriculum; nine weekly 40–60 min lessons; Kuyken et al., 2013) was less intensive compared to the programme used in our trial (eight weekly sessions of 90–100 min). Third, in the MYRIAD trial, the MT was delivered by school teachers, whereas MTs in the current trial were offered by certified and experienced trainers. However, in the light of these study design differences, the current findings can be considered as complementary to the MYRIAD trial results.

Taken together, the evidence suggests that universal school-based MTs, at least as currently typically implemented, may not be the most efficient path towards overall better mental health among adolescents. There are several micro-, meso- and macro-level factors that need to be considered in future innovation and research.

One potentially crucial micro-level factor is the overall low frequency of home practice, as observed in the current study. This finding is in line with other studies in the field (Tudor et al., 2022). However, home practice has been emphasized as an integral part and necessary condition of MBCT and MBSR programmes in order to develop mindfulness skills (Parsons, Crane, Parsons, Fjorback, & Kuyken, 2017). Empirical support for the positive association, albeit rather small, between the extent of (formal) practice and intervention effectiveness was found in adult samples, especially for novice practitioners (e.g., Levi, Shoham, Amir, & Bernstein, 2021; Parsons et al., 2017), and in school settings (Tudor et al., 2022). In other words, limited engagement in home practice by the majority of the adolescents may be one reason for the absence of substantial improvements over time. Exploratory analyses lend preliminary support for the role of active engagement for the MT to exert its full effect and initiate symptom improvement. However, future fully-powered studies are needed, which ideally map engagement with the MT in a systematic and fine-grained way to draw robust conclusions (e.g., daily reporting of level of home practice, types of exercises most frequently practiced with).

One way to increase engagement in home practice, and in the MT in general, involves adapting MTs to reach an optimal fit with adolescents' needs and preferences (cf. Co-design; e.g., Foulkes & Stapley, 2022; Kuyken et al., 2022). For instance, adolescents’ answers to the open questions in the current study already imply the need for consideration of some practical changes (e.g., higher frequency but shorter session duration, more variation in types of exercises). Such changes can be implemented easily, probably even without interference with underlying working mechanisms. However, and more challenging, there also appears to be a considerable diversity of individual experiences/attitudes towards the MT (e.g., intrinsic motivation or interest). This forms a more fundamental barrier which can probably only be dealt with by moving towards more differentiation (or diverging from the universal approach).

A barrier at the meso-level may be a suboptimal class social climate or the presence of unhelpful group dynamics. As sharing private thoughts and feelings with classmates is a core aspect of MT, a crucial condition to be met is that adolescents actually feel sufficiently comfortable and safe (Foulkes & Stapley, 2022). Healthy social interactions have indeed been suggested to be necessary for the motivation to learn and develop intrapersonal higher-order mental processes, like mindfulness (Roeser et al., 2023). In that way, a suboptimal class social climate or unhealthy group dynamics may jeopardize the potential beneficial impact of the MT or, exceptionally, even initiate an opposite effect (e.g., increased feelings of distress). For instance, given the prevalence of bullying at school (Inchley et al., 2020), a number of adolescents may even experience sharing private information with peers as stressful. In contexts characterised by rather unhealthy group dynamics, an intentional focus on developing interpersonal skills and/or a caring classroom environment might be a crucial prerequisite for intrapersonal skills to be developed (e.g., via MT). In contrast, in case of relatively healthy social dynamics, mindfulness-based interventions may even improve social relationships (Donald et al., 2019; López-González, Amutio, & Herrero-Fernández, 2018). This effect may be partly explained by the facilitating role of the mindfulness trainer who attempts to establish a safe group climate (e.g., setting clear boundaries) to get in touch with emotional vulnerabilities (Cormack et al., 2018). Related to the group context in which the MT takes place, negative attitudes towards the MT of one or a few adolescents can be easily spread across the group and undermine its potential overall beneficial impact (Thompson & Gauntlett-Gilbert, 2008).

Factors related to the broader school context (meso-level) may also act as potential barriers, as suggested by the growing literature on the facilitating or frustrating impact of school contexts on the implementation of evidence-based programmes in schools (Domitrovich, Li, Mathis, & Greenberg, 2019; Nguyen et al., 2021). School context/climate may even be influenced by factors at the macro-level. For instance, in the current study, it may be that a specific interplay between school context and the macro-context (i.e., COVID-19) has attenuated the potential (small) effect of the MT. That is, the COVID-19 period during which the study took place may have faced school teams and management with additional organizational challenges (vs. pre-COVID times). This may have reduced resources at school (e.g., time, opportunity to speak to adolescents in real life) to motivate adolescents, especially those who were not intrinsically motivated, to fully engage in the MT.

These examples related to the individual, the group and school context illustrate potential invisible barriers for the implementation and therefore effectiveness of universal school-based MT. Ideally, future studies should systematically focus on the moderating role of such factors across levels of analyses (e.g., intrinsic motivation, class and school context, culture, socio-economic climate). Learning more about moderating factors across levels (for an overview, see Tudor et al., 2022) is necessary to gradually move closer to an answer to the question ‘What works, for whom, and how?’ (Kuyken et al., 2022).

The multitude of possible moderators and the complex interplay of factors across levels may also imply the need for reconsidering universal school-based MTs. For instance, a parallel line of research could focus on the effectiveness of more pragmatic and potentially more efficient approaches to the implementation of MT in school settings. One possibility may be to first offer general introductory sessions. Instead of working with pre-existing classes of adolescents, only adolescents who are interested in and intrinsically motivated will opt into the MT. This may increase the probability of full and long-lasting engagement in the MT (incl. Home practice, which might help to overcome a potentially crucial barrier at the individual level). Positive attitudes and experiences with the MT may eventually diffuse among other adolescents and nudge initially reluctant adolescents towards following the MT.

The abovementioned findings and suggestions for future studies should be interpreted taking into consideration the following limitations. First, although a multi-method approach was adopted (ESM, SRQs, open questions), findings were exclusively based on adolescents’ self-report and may be influenced by for instance memory bias or social desirability.

Second, treatment protocol adherence (e.g., deviations due to external factors) was not formally evaluated. However, it is highly unlikely that the way the MT was delivered accounts for the lack of significant effects in the current study. First, as mentioned above, the MT was delivered by certified and experienced mindfulness trainers with a clinical background, and regular supervision was organised during the trial. This should be considered as an absolute strength of the study, as greater benefits follow from school-based MT delivered by optimally trained facilitators than by non-experts (Tudor et al., 2022). Of particular relevance in the current study, mindfulness trainers could rely on their experience to flexibly respond to particular difficulties related to the COVID-19 period (e.g., unstable school climate, flexible planning of sessions), while still adhering to the training protocol. Second, although a minimal number of MT sessions was delivered via synchronous online communication, the majority of the sessions took place at school (see Supplementary Material). Nevertheless, online MTs have been found to be effective in a broad range of populations (e.g., Messer, Horan, Turner, & Weber, 2016; Nadler, Carswell, & Minda, 2020; Sommers-Spijkerman, Austin, Bohlmeijer, & Pots, 2021) and to have an added value in combination with regular treatment in different subgroups of patients (e.g., Compen et al., 2018; Kladnitski et al., 2020; Segal et al., 2020). Moreover, the aforementioned evidence is mainly based on MT delivered through asynchronous communication (i.e., not in real-time, using pre-recorded audio files). Since synchronous online communication in our study allowed real-time interaction between the trainer and the participants, synchronous communication can be assumed to be at least as effective as asynchronous communication.

Third, considerable drop-out rates due to low ESM compliance in the MT group may reflect a too high study burden for some participants. Inadvertently, this may have influenced participants’ attitude towards mindfulness or may have partially undone its beneficial impact. Related to the ESM, reward was made partially conditional on compliance rates during the course of the trial to increase ESM compliance. On the one hand, compensating participants conditional on compliance rates is very common in ESM research in adolescents (e.g., Cushing, Bejarano, Mitchell, Noser, & Crick, 2018; Glenn et al., 2020; Pouwels, Valkenburg, Beyens, van Driel, & Keijsers, 2021) and it has even been recommended to increase compliance in this age group (van Roekel, Keijsers, & Chung, 2019). On the other hand, conditional incentives may have inadvertently impacted the quality of the data (Mölsa, Lax, Korhonen, Gumpel, & Söderberg, 2022), resulting in impaired validity and/or reliability. However, as our app settings prevented *backfilling* (i.e., fill in all the questionnaires at once, after the required time; Dejonckheere & Erbas, 2021), the potential detrimental impact of conditional rewarding was considerably reduced.

Fourth, a small group of adolescents in the MT prematurely withdrew from the study during the course of the MT. Although no formal adverse events were registered, involvement of (mildly) adverse experiences in study withdrawal cannot be ruled out. Future studies in adolescents should systematically include sensitive measures on meditation-related adverse events throughout the training programme. So far, research in adults has already shown mediation-related adverse events rates similar to other psychological treatments, irrespective of the presence of prior mental health problems (Britton, Lindahl, Cooper, Canby, & Palitsky, 2021; Farias, Maraldi, Wallenkampf, & Lucchetti, 2020). Alternatively, study withdrawal may simply be related to a mismatch between the training and individual preferences of adolescents. The broad variety of attitudes towards the MT in the current sample, in line with other research in the field (Montero-Marin et al., 2022; Montero-Marin et al., 2023), lends some first support for this alternative explanation.

Fifth, the outbreak of and ongoing COVID-19 pandemic might have influenced adolescents’ emotional states and response styles or their psychological flexibility to actually learn and implement alternative ways of relating to their inner and outer environment. However, no indications of the immediate and short-term impact of the COVID-19 pandemic, like higher levels of stress or anxiety (Cohen et al., 2021; Degenhardt, 2022; Panchal et al., 2021; Samji et al., 2022; Theberath et al., 2022) were found in this study sample. It should be noted that, in the opposite direction, the presence of floor effects does probably not yield a sufficient explanation for the absence of significant effects in the current study either. Baseline depressive symptomatology reached a similar level as in Raes et al. (2014), in which small to moderate depressive symptom reductions were found over time (statistically and clinically significant). Nevertheless, future studies could additionally focus on the impact of (universal school-based) MT on levels of negative and positive affect (related to respectively emotional distress and anhedonia). This might further increase sensitivity for detection of affective changes as a result of MT delivered in community samples of adolescents (vs. changes in symptomatology).

Sixth, sample size planning was based on our experience with ESM data. Future research should rely on recently developed, more accurate tools for sample size planning for multilevel linear regression models (e.g., Lafit et al., 2021). Given the pioneering ESM approach in this research field, sample size could not be compared with prior studies. However, when taking into account all SRQs available, the sample size of the current study exceeds those of previous studies that found evidence for the beneficial impact of universal school-based MT (e.g., Raes et al., 2014). This could be considered as counter-evidence for the lack of power (for the SRQs) as an explanation for the absence of substantial effects in the current study.

To conclude, this cluster RCT did not find support for a significant positive impact of universal school-based MT on emotional distress and levels of anhedonia among adolescents. No direct impact of the MT on the hypothesized underlying mechanisms (treated as outcomes) was found either. Further research should prioritise learning more about the impact of moderators and implementation factors across levels of analysis. For instance, potential (nuanced) positive effects of school-based MTs for some adolescents may now be overshadowed by the absence of significant changes for others. Additionally, alternative ways of delivering MT at schools (e.g., intrinsically motivated adolescents as primary target group) should be investigated in parallel. These alternative formats may be associated with less potentially attenuating factors compared to universal programmes, and therefore be more pragmatic and efficient ways to improve adolescents’ mental health.

## Funding

This research was supported by the Research Foundation - 10.13039/501100011878Flanders (FWO-10.13039/501100011878Vlaanderen) under a red Noses grant (G049019N). KVDG and PK were supported by the 10.13039/501100004040KU Leuven Research Council grant C14/19/054; MS was supported by the Research Council 10.13039/501100004040KU Leuven (project C14/19 to E. Ceulemans and P. Kuppens); MK was supported by the Research Foundation – 10.13039/501100011878Flanders (FWO-10.13039/501100011878Vlaanderen) under a Red Noses grant (G049019N) and under a PhD fellowship (11I1622N); WK's work was supported by the 10.13039/100010269Wellcome Trust (WT104908/Z/14/Z and WT107496/Z/15/Z).

## CRediT authorship contribution statement

**Liesbeth Bogaert:** Data curation, Formal analysis, Investigation, Methodology, Visualization, Writing – original draft, Writing – review & editing. **Katleen Van der Gucht:** Supervision, Conceptualization, Methodology, Writing – review & editing. **Peter Kuppens:** Supervision, Conceptualization, Methodology, Writing – review & editing. **Merle Kock:** Formal analysis, Methodology, Writing – review & editing. **Marieke J. Schreuder:** Formal analysis, Methodology, Writing – review & editing. **Willem Kuyken:** Supervision, Writing – review & editing. **Filip Raes:** Supervision, Conceptualization, Methodology, Funding acquisition, Writing – review & editing.

## Declaration of competing interest

The authors declare that they have no known competing financial interests or personal relationships that could have appeared to influence the work reported in this paper

## Figures and Tables

**Table A1:** Items of the Non-Acceptance and Suppression of Negative Emotions Scale (Raes, 2019; unpublished) and Results of Principal Component Analysis

Label	Item	Component 1 suppression	Component 2 non-acceptance
nasnes_1	I have difficulties to tolerate negative feelings.	−.178	**−.915**
nasnes_2	I find it difficult to accept negative feelings.	−.010	**−.874**
nasnes_3	I try to avoid negative feelings.	**.834**	.107
nasnes_4	I try not to allow negative feelings.	**.875**	.091
nasnes_5	I find it hard to accept negative feelings.	.121	**−.809**
nasnes_6	I prefer negative feelings to disappear as soon as possible.	**.680**	−.107
nasnes_7	I try to repress negative feelings.	**.749**	−.101
nasnes_8	I find it hard to open up for negative feelings.	.292	**−.599**
nasnes_9	I try to suppress negative feelings.	**.656**	−.187
nasnes_10	I find it difficult to allow negative feelings.	.358	**−.625**

*Notes.* (a) The original scale consists of Dutch items. (b) Principal component analysis (oblique rotation) yielded sufficient evidence to retain the two theoretically presumed subscales, namely non-acceptance and suppression. Factor loadings with a value > |0.40| were considered as high.

**Table A2:** Items of the Adapted 13-item Social Expectancies to experience Depression and Anxiety Scale (SEDAS; Bastian et al., in preparation; McGuirk et al., 2018)

Label	Item
	Negative Thoughts/Emotions
sedas_neg_1	I think my classmates accept people who feel depressed/anxious as normal. *
sedas_neg_2	I think in my class it is socially acceptable to feel depressed/anxious. *
sedas_neg_3	My classmates generally expect people NOT to feel depressed/anxious.
sedas_neg_4	My classmates expect me NOT to feel depressed/anxious.
sedas_neg_5	My classmates like me less when I feel depressed/anxious.
sedas_neg_6	I think that my classmates tend to place a lot of pressure on each other NOT to feel depressed/anxious.
sedas_neg_7	I think that most of my classmates tend to disapprove of people who feel depressed/anxious.
sedas_neg_8	Most of my classmates would see people who feel depressed/anxious as failing in life.
sedas_neg_9	Overall, my classmates are comfortable with those who feel depressed/anxious. *
sedas_neg_10	Generally speaking, my classmates prefer not to associate with me when I feel depressed/anxious.
sedas_neg_11	I feel a great deal of pressure from my classmates NOT to feel depressed/anxious.
sedas_neg_12	When my classmates see me as depressed/anxious, they probably think I am a failure.
sedas_neg_13	I think most of my classmates value me just the same even when I feel depressed/anxious. *

*Notes.* (a) The original scale consists of Dutch items. (b) Reverse scored items are indicated by *. (c) For the subscale on ‘negative thoughts’, a high total score reflects high perceived pressure from classmates NOT to experience/express negative feelings/emotions.

**Table:** 

	Control						Intervention (MT)					
	T1		T2		T3		T1		T2		T3	
	*n*	%	*n*	%	*n*	%	*n*	%	*n*	%	*n*	%
Extremely severe (28+)	6	4.41	14	10.29	4	2.94	11	11.58	7	7.37	9	9.47
Severe (21–27)	4	2.94	6	4.41	9	6.62	6	6.32	3	6	9	9.47
Moderate (14–20)	27	19.85	24	17.65	28	20.59	13	13.68	14	14.74	12	12.63
Mild (10–13)	12	8.82	20	14.71	26	19.12	12	12.63	12	12.63	5	5.26
Normal ( ≤ 9)	87	63.97	72	52.94	69	51.74	53	55.79	59	62.11	60	63.16

*Note.* Categories are based on the DASS-21 subscale for depressive symptoms (Lovibond & Lovibond, 1995).

**Table C1:** Main and Interaction Fixed Effects of Multilevel Model Estimating Main Outcomes (H1 and H2; ESM Data) With Bootstrapping

	Anhedonia (H1)					
	Difficulties to Enjoy		No Expected Pleasure		Not Feeling Happy	
	Est.	95% CI	Est.	95% CI	Est.	95% CI
Intercept	−0.20	[-0.36;-0.07]*	−0.20	[-0.36; 0.06]*	−0.15	[-0.30; 0.018]
Age	0.11	[0.03; 0.17]*	0.09	[0.02; 0.16]*	0.05	[-0.02; 0.12]
Gender	0.24	[0.05; 0.42]*	0.11	[-0.06; 0.27]	0.13	[-0.03; 0.29]
T2	0.02	[-0.05; 0.10]	0.20	[0.13; 0.28]*	−0.15	[-0.27;-0.03]*
T3	0.04	[-0.06; 0.12]	0.17	[0.06; 0.30]*	0.15	[0.03; 0.31]*
Condition	0.06	[-0.12; 0.23]	0.01	[-0.14; 0.18]	0.07	[-0.09; 0.31]
T2:Condition	−0.03	[-0.15; 0.09]	−0.06	[-0.22; 0.09]	−0.02	[-0.25; 0.15]
T3:Condition	−0.11	[-0.23; 0.04]	−0.04	[-0.22; 0.13]	−0.04	[-0.26; 0.10]
	Emotional Distress (H2)					
	Depressive		Anxious		Stressed	
	Est.	95% CI	Est.	95% CI	Est.	95% CI
Intercept	−0.14	[-0.34; 0.03]	−0.46	[-0.57; −0.36]*	−0.50	[-0.57;-0.31]*
Age	0.08	[0.003; 0.16]*	0.12	[0.04; 0.21]*	0.07	[-0.004; 0.15]
Gender	0.22	[0.06; 0.39]*	0.41	[0.26; 0.54]*	0.52	[0.36; 0.68]*
T2	−0.07	[-0.23; 0.08]	0.18	[0.10; 0.27]*	0.12	[0.02; 0.20]*
T3	0.01	[-0.15; 0.16]	0.21	[0.11; 0.30]*	0.05	[-0.05; 0.16]
Condition	0.09	[-0.12; 0.38]	0.14	[-0.003; 0.32]	0.08	[-0.09; 0.24]
T2:Condition	−0.04	[-0.26; 0.18]	−0.03	[-0.18; 0.08]	0.04	[-0.12; 0.23]
T3:Condition	−0.06	[-0.27; 0.16]	−0.08	[-0.23; 0.08]	0.004	[-0.12; 0.19]

*Note.* If zero is not included in the 95% CI the estimate can be considered as significant (*).

**Table C2:** Main and Interaction Fixed Effects of Multilevel Model Estimating Outcomes Putative Mediators (H1, H2, H3) With Bootstrapping

	Dampening (H1)		Suppression NA (H2)		Non-Acceptance NA (H2)		Pressure no NA (H3)	
	Est.	95% CI	Est.	95% CI	Est.	95% CI	Est.	95% CI
Intercept	−0.13	[-0.31; 0.08]	0.04	[-0.14; 0.23]	−0.10	[-0.30; 0.08]	−0.13	[-0.28; 0.03]
Age	0.09	[-0.00; 0.18]	−0.02	[-0.12; 0.05]	−0.03	[-0.10; 0.05]	0.09	[0.004; 0.16]*
Gender	0.18	[-0.00; 0.38]	0.15	[-0.02; 0.32]	0.14	[0.00; 0.33]*	−0.01	[-0.20; 0.14]
T2	−0.14	[-0.29;-0.05]*	−0.19	[-0.27;-0.11]*	−0.12	[-0.23; 0.02]	−0.09	[-0.18; 0.008]
T3	0.07	[-0.05; 0.21]	−0.34	[-0.47;-0.24]*	0.12	[-0.00; 0.23]	0.30	[0.18; 0.44]*
Condition	0.10	[-0.11; 0.34]	0.11	[-0.08; 0.29]	−0.01	[-0.24; 0.15]	0.16	[-0.05; 0.35]
T2:Condition	−0.03	[-0.20; 0.17]	0.02	[-0.16; 0.17]	0.04	[-0.11; 0.23]	0.02	[-0.14; 0.16]
T3:Condition	−0.11	[-0.30; 0.10]	−0.01	[-0.23; 0.18]	−0.06	[-0.25; 0.10]	−0.10	[-0.34; 0.11]

*Note.* If zero is not included in the 95% CI the estimate can be considered as significant (*).

**Table D1:** Main and Interaction Fixed Effects of Multilevel Model Estimating Main Outcomes (H1, H2; SRQ Data) With Bootstrapping

	Anhedonia (H1)		Emotional Distress (H2)					
			Depressive		Anxious		Stressed	
	Est.	95% CI	Est.	95% CI	Est.	95% CI	Est.	95% CI
Intercept	−0.26	[-0.48;-0.03]*	−0.36	[-0.54;-0.11]*	−.050	[-0.64;-0.33]	−0.58	[-0.74;-0.40]*
Age	0.07	[-0.05; 0.22]	0.05	[-0.05; 0.17]	−0.04	[-0.13; 0.07]	0.06	[-0.06; 0.17]
Gender	0.24	[0.01; 0.50]*	0.33	[0.13; 0.58]*	0.52	[0.32; 0.73]*	0.62	[0.43; 0.81]*
T2	0.13	[-0.02; 0.26]	0.19	[0.04; 0.32]*	0.25	[0.10; 0.38]*	0.15	[0.01; 0.30]*
T3	0.17	[0.02; 0.31]*	0.12	[-0.07; 0.27]	0.22	[0.06; 0.34]	0.13	[-0.02; 0.32]
Condition	0.08	[-0.13; 0.32]	0.26	[-0.03; 0.51]	−0.01	[-0.19; 0.20]	0.09	[-0.17; 0.30]
T2:Condition	−0.22	[-0.45;-0.04]*	−0.44	[-0.61;-0.22]*	−0.05	[-0.27; 0.18]	0.08	[-0.15; 0.28]
T3:Condition	−0.22	[-0.40;-0.04]*	−0.25	[-0.48; 0.04]	−0.08	[-0.28; 0.23]	−0.02	[-0.29; 0.23]

*Note.* If zero is not included in the 95% CI the estimate can be considered as significant (*).

**Table D2:** Main and Interaction Fixed Effects of Multilevel Model Estimating Outcomes Putative Mediators (H1, H2, H3; SRQ Data) With Bootstrapping

	Dampening (H1)		Suppression NA (H2)		Non-Acceptance NA (H2)		Pressure non-expression/experience of NA (H3)	
	Est.	95% CI	Est.	95% CI	Est.	95% CI	Est.	95% CI
Intercept	−0.19	[-0.41; 0.04]	0.14	[-0.07; 0.43]	−0.16	[-0.35; 0.15]	0.28	[0.10; 0.44]*
Age	0.05	[-0.04; 0.16]	−0.04	[-0.14; 0.08]	0.03	[-0.07; 0.14]	0.09	[-0.01; 0.20]
Gender	0.36	[0.12; 0.60]*	0.14	[-0.13; 0.40]	0.43	[0.22; 0.66]*	−0.06	[-0.24; 0.16]
T2	−0.11	[-0.23; 0.01]	−0.23	[-0.39;-0.08]*	−0.16	[-0.34; 0.01]	−0.26	[-0.35;-0.11]*
T3	−0.13	[-0.25;-0.008]*	−0.290	[-0.46;-0.11]*	−0.14	[-0.33; 0.03]	−0.34	[-0.45;-0.20]*
Condition	0.08	[-0.11; 0.33]	−0.10	[-0.39; 0.09]	−0.01	[-0.25; 0.22]	−0.10	[-0.30; 0.14]
T2:Condition	−0.07	[-0.24; 0.12]	−0.02	[-0.26; 0.20]	−0.08	[-0.31; 0.17]	0.01	[-0.17; 0.16]
T3:Condition	−0.06	[-0.24; 0.14]	−0.15	[-0.40; 0.05]	−0.19	[-0.41; 0.06]	−0.12	[-0.34; 0.08]

*Note.* If zero is not included in the 95% CI the estimate can be considered as significant (*).

## Data Availability

Coded, pseudonymized dataset will be uploaded in a csv format to Open Science Framework in a restricted access repository.

## Footnotes

1 Details about the timing of the multiple rounds of data collection are added in the Supplementary Material as well as information about the interference of COVID-19 across rounds of data collection.

2 At some schools, the follow-up interval had to be shortened (most extremely to two months) due to exam periods at the end of the academic/school year.

3 Additional background information about the key objectives of the MT and the content per session are provided as supplementary material.

4 https://ppw.kuleuven.be/leuven-mindfulness-centre/Open-Source.

5 Other measures (secondary endpoints) were not reported in this paper but can be found in the preregistration.

6 Information about the pre-assessment phase (e.g., app installation) is added as supplementary material.

7 Results of the drop-out analyses are added as supplementary material.

8 Visual presentation of the results of the main analyses for both the outcome measures and the putative mediators, as well as random effects, based on the ESM and SRQ data, can be found in the Supplementary Material.
