# Supplementary material for: The effect of universal school-based mindfulness on anhedonia and emotional distress and its underlying mechanisms: A cluster randomised controlled trial via experience sampling in secondary schools
Source: Behav Res Ther. 2023 Oct;169:104405. doi: 10.1016/j.brat.2023.104405 (PMC10938062; doi:10.1016/j.brat.2023.104405)
Supplement: Multimedia component 1 [file mmc1.docx]

## Supplementary Material

## Timing Data Collection and COVID-19 Interference

The first round of data collection (three schools) started in January 2020. Post-intervention and follow-up assessment took place in March 2020 and May/June 2020. The second round of data collection (one school) started in September 2020 and ended in February 2021 (with the post-intervention assessment in November/December 2020). Two schools took part in the third round of data collection, which started in January 2021. Post-intervention and follow-up assessment of this round were conducted in March 2021 and May/June 2021 respectively. The fourth round consisted of three schools. The first, second, and third assessment took place in September 2021, November/December 2021, and February 2022 respectively. The final and fifth round of data collection (four schools) started in January 2022 and ended in May/June 2022 (with the post-intervention assessment in March 2022). Data collection was ended as soon as the required sample size (based on ESM compliance) was reached.

Data collection had to be prolonged (although it was never interrupted) due to interference of COVID-19. First, the high organisational burden put on schools due to frequently changing COVID-19 measures caused some schools that were initially interested to deter from participation (before the start of data collection). Consequently, not all rounds of data collection were run at full capacity, and two schools with class groups of less than 12 participants were allowed to take part in the study. Second, particularly in the first cohort, considerable drop-out rates were observed at post-intervention assessment (see *Results*). This assessment point took place immediately after enforcement of the first lockdown in Belgium (including physical closure of schools in March 2020), which may explain the high drop-outs at this stage.

Concerning the MT, in case of physical closure of the participating schools in the course of the MT (cf. COVID-19 measure), synchronous (i.e., real-time) online mindfulness sessions substituted for the regular in-class sessions. MT sessions were delivered via an online synchronous communication platform that included the option to display all participants in a gallery view (i.e., most satisfactory approximation of real class climate), and the use of breakout rooms (i.e., sharing experiencing with one another in small groups). Regardless of this format-related modification, the number and content of the sessions remained unaltered. Overall, the number of times we had to resort to online training sessions was kept to a minimum (i.e., maximal two times in one school, and in many schools no online sessions were needed). Due to organisational difficulties and continuously changing circumstances (e.g., blended education), some intervention groups exceptionally received two MT sessions per week (after exploring all other alternative options). Also, MT sessions were scheduled in a rather flexible way (i.e., the exact moment of the weekly session rotated). Irrespective of these format- and planning-related changes, all intervention groups were offered all essential components of the MT as planned.

Finally, concerning the assessment points, some SRQs had to be administered through a Qualtrics survey (GDPR-proof system) (e.g., due to physical absence of participants). In particular, participants received an email inviting them to complete the SRQs online. Access was granted by clicking an anonymous weblink, and similar to pen-and-paper administration, participants filled in their pseudonym ID. The content of the questionnaires was identical to the paper-pencil version, and participants could also skip or reconsider questions. If individual participants were absent (e.g., illness or COVID-19 isolation), participants were invited (if possible) to complete the SRQs, either via an online survey link or by manually completing the SRQs that were sent as a digital attachment. All the other assessments took place at school, via pen-and-paper SRQs. At one school (Round 4), the post-intervention administration was led by a school teacher instead of by the researcher due to strict COVID-19 measures at the time of administration (i.e., no external visitors allowed at school). For the sake of uniformity (i.e., instructions and procedure followed by the researcher), a written form with clear instructions was offered to the teacher in advance.

**Description of the Intervention**

Key objectives of the MT used in our study were (1) to increase awareness of one’s present moment experience, and (2) to teach an attitude of openness and acceptance (non-judging) towards one’s experience(s). This open and accepting attitude changes the person’s relationship with the experience towards a detached and non-reactive orientation. Participants learn to recognize entanglement with one's thoughts and emotions and there is an increased understanding of one’s spontaneous and habitual reactions. If adolescents successfully adopt these skills, their negative emotions and cognitions will no longer be reinforced, creating the opportunity to deal with problematic thoughts and feelings. The programme uses extensive training in mindfulness meditation practice, and mindfulness meditation practice is employed as the central foundational methodology (Crane et al., 2017; Santorelli, 2021). The core elements of the 8-week MT are summarised in Table S1.

Throughout the MT, mindfulness trainers frequently (i.e., each session) stressed the importance of practising mindfulness skills on a regular (preferably daily) basis to be able to gradually experience changes. In other words, adolescents were regularly reminded about the importance of practising their skills on a daily basis (on average 15 minutes). In addition, each session adolescents were invited to share their individual experiences with practising mindfulness between the sessions. Finally, adolescents were provided with the You-Mind app with audio files of guided exercises (including some FAQs and information videos) to further support practice at home.

**Table S1**

*Core Elements of the MT, Related Exercises in the You-Mind app to Support Practice at Home, and Tips to Practise in an Informal Way Between Sessions*

| Session | Content |
| --- | --- |
| 1 | In-session: discovering that we function on automatic pilot and kind attention to the body - brief grounding exercise, spotlight metaphor of attention, ground rules, intentions, pause exercise, eating exercise, body scan |
|  | You-Mind app: guided exercise (pause exercise, body scan), FAQ and info (spotlight metaphor of attention); Tip to practise (informal): mindful eating |
| 2 | In-session: gently dealing with barriers while practicing and befriending the breath - body scan, monkey-mind and puppy metaphor of thoughts, pause exercise, anchor exercise, entanglement of thoughts, feelings, body-sensations |
|  | You-Mind app: guided exercise (body scan, breathing focus), FAQ and info (monkey-mind and entanglement of thoughts); Tip to practise (informal): Noticing obstacles while practicing and entanglement of thoughts/feelings/sensations related to a pleasant experience, conduct routine activities with attention (e.g., brushing teeth) |
| 3 | In-session: gently learning to work with personal limits and befriending the breath - mindful movement, pleasant experience and entanglement (thoughts-feelings-sensations), 3-minute breathing space  You-Mind app: guided exercise (personal limits, breathing space); Tip to practise (informal): mindful walking during the day, entanglement of thoughts/feelings/sensations related to a unpleasant experience |
| 4 | In-session: discovering that we can choose how to respond by opening gently to experience - sitting meditation, unpleasant experience and entanglement (thoughts-feelings-sensations), pause and barometer exercise, 3-minute breathing space as responding tool  You-Mind app: guided exercise (breathing space, hourglass); Tip to practise (informal): responding instead of reacting, hourglass, kind wishes related to unpleasant experience |
| 5 | In-session: gently being with what is difficult - pause and barometer exercise, sitting meditation being with what is difficult + half way review - recommitting personal intentions, extended 3-minute breathing space as responding tool  You-Mind app: guided exercise (barometer, pause exercise); FAQs and info (insights barometer); Tip to practise (informal): practise with pause and barometer exercise if test or schoolwork, hourglass as a response to unpleasant experience |
| 6 | In-session: learning to accept that thoughts are not facts - pause and barometer exercise, sitting meditation (sounds and thoughts), extended breathing space, gently experiencing the power of thoughts, mountain meditation |
|  | You-Mind app: guided exercise (sounds/feelings/thoughts, mountain); Tip to practice (informal): thieves in the nights (metaphor power of thoughts) |
| 7 | In-session: taking care of ourselves - pause and barometer exercise, mindful movement, extended breathing space plus action step, spiral signature and stress signals, loving kindness exercise  You-Mind app: guided exercise (personal limits); Tip to practice (informal): in case of signals of tension or stress, hourglass exercise with caring action |
| 8 | In-session: going further, beyond fear, pause and barometer, choiceless awareness meditation, spiral signature and generating a personal list of practices - reviewing personal action plans, review course, loving kindness meditation |
|  | You-Mind app: guided exercise (choiceless awareness, kind wishes); Tip to practice (informal): continue practicing on a daily basis (max. 15 minutes/day) |

**Pre-Assessment Procedure**

ESM data were collected via our study’s mobile application (the You-Mind app) that participants downloaded on their personal smartphones. Instructions to install the You-Mind app were sent via email to adolescents before the first assessment of the SRQs. In case of difficulties with app installation, the researcher provided assistance immediately after completion of the baseline SRQs in class. In addition, adolescents received a leaflet with information about the ESM procedure that was about to follow (usually) the next day (e.g., dates and time window during which they would receive beeps, (aimed) number of beeps (to be completed), contact information of the researcher in case technical problems arose). For the post-training and follow-up assessment, general instructions about the ESM procedure were repeated orally after administration of the SRQs, as well as in a written form (leaflet). Identical information was sent to the school management and/or the contact person at school (responsible for the coordination of the project at school), accompanied by the request to forward the information to the team of teachers of the participating classes. In that way, all parties, directly or indirectly involved in the study, were fully informed about the timing and practical study specifics (e.g., beeps during lessons). This also allowed teachers to regularly emphasize the importance of ESM compliance and, subsequently, stimulate participants to complete as many ESM notifications as possible.

Together with the instructions to install the You-Mind app, each participant received a unique pseudonym ID, which was linked to participants’ individual login token they had to fill in during app installation. The pseudonym ID was composed of the first letters of the city location of the school, a unique meaningless number referring to the class group (often either ‘1’ or ‘2), and a participant number (e.g., h1_15 or d2_05). This enabled us to link ESM data and SRQ data, as participants were asked to fill in the same ID on the first page of their booklet with SRQs (i.e., no name or other potential personal identifiers).

**Drop-out Analyses**

Drop-out based on the ESM data (i.e., insufficiently high compliance) was substantially higher than based on the SRQs. Low ESM compliance rates may be partly explained by technical difficulties. However, in fact only a few participants contacted the researchers to report technical difficulties. So, more plausibly, low ESM compliance rates were caused by an interplay of other factors at an individual and contextual level (e.g., low motivation, attitude towards the study, absence of support by teachers, high burden). Factors related to the challenging macro-context (e.g., unstable (school) climate due to COVID-19) may have amplified the influence of such factors.

Missingness due to low ESM compliance (in the presence of SRQ data) appeared not to be associated with levels of depressive symptoms reported via the SRQs at the same or previous assessment point. So, it is unlikely that ESM missingness resulted from the ESM procedure being too burdensome in the presence of emotional difficulties. However, regarding group membership, significantly higher drop-out rates were observed for the intervention group compared to the control group at post-intervention (*X*²(1, *N* = 455) = 8.198, *p* = .004), and at follow-up (*X*²(1, *N* = 455) = 11.638, *p* < .001). Regarding drop-out due to the absence of SRQ only, drop-out at post-intervention was not related to group membership (*X*²(1, *N* = 455) = 0.70, *p* = .40). Drop-out at follow-up was only marginally significantly higher for the intervention group compared to the control group (*X*²(1, *N* = 455) = 4.00, *p* = .05). For ESM missingness (i.e., no data or too low ESM compliance), significant associations with group membership were found both at post-intervention and at follow-up (respectively, *X*²(1, *N* = 455) = 10, *p* = .002; *X*²(1, *N* = 455) = 15, *p* < .001). So, higher ESM missingness in the intervention group may be linked with the higher effort related to participation in this study arm (i.e., three assessment points and the 8-week intervention).

**Visual Representation of Main and Interaction Fixed Effects of Multilevel Model Estimating Outcomes (ESM Data and SRQ Data)**

**Figure S1**


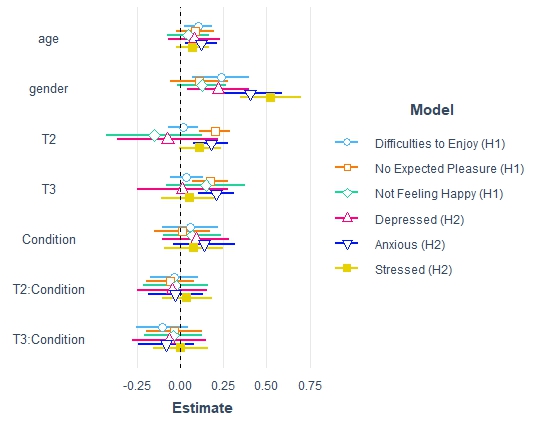
*Main and Interaction Fixed Effects of Multilevel Model Estimating Main Outcomes (H1 and H2; ESM Data)*

*Note.* If the 95% CI includes zero, then then estimate is considered as insignificant.

**Figure S2**

*Main and Interaction Fixed Effects of Multilevel Model Estimating Putative Mediators (H1, H2, H3; ESM Data)*


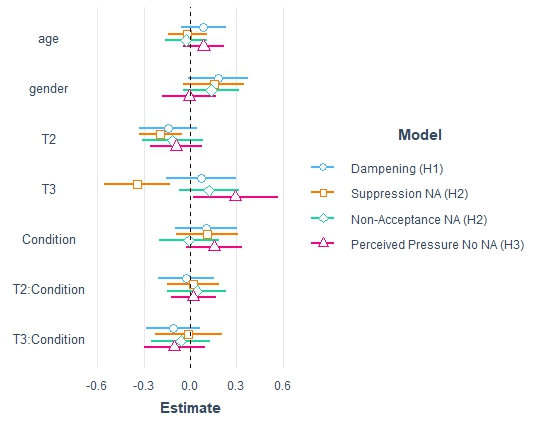


*Note*. If the 95% CI includes zero, then then estimate is considered as insignificant.

**Figure S3**

*Main and Interaction Fixed Effects of Multilevel Model Estimating Main Outcomes (H1 and H2; SRQ Data)*


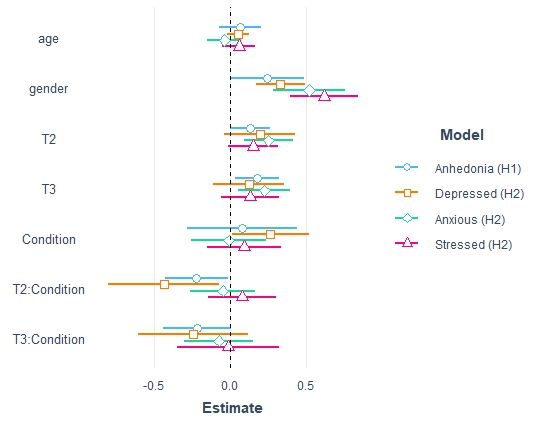


*Note.* If the 95% CI includes zero, then then estimate is considered as insignificant; No correction for multiple testing was applied.

**Figure S4**

*Main and Interaction Fixed Effects of Multilevel Model Estimating Putative Mediators (H1, H2, H3; SRQ Data)*


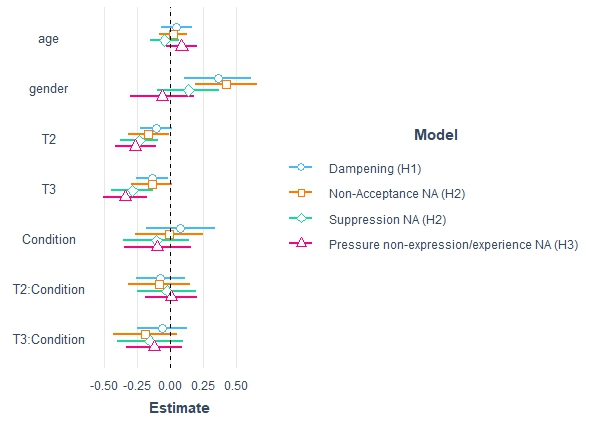


*Note.* If the 95% CI includes zero, then then estimate is considered as insignificant.

**Random Effects of Multilevel Models Estimating Main Outcomes and Putative Mediators (ESM Data and SRQ Data)**

**Table S2**

*Random Effects of Multilevel Models Estimating Main Outcomes (H1 and H2; ESM Data)*

| \|  \|  \| Anhedonia (H1) \| \| \| \| \| \| \| \| \| \| \| \| \| \| \| --- \| --- \| --- \| --- \| --- \| --- \| --- \| --- \| --- \| --- \| --- \| --- \| --- \| --- \| --- \| --- \| \|  \|  \| Difficulties to Enjoy \| \| \| \| \| No Expected Pleasure \| \| \| \| \| Not Feeling Happy \| \| \| \| \| Groups \| Variable \| *Var* \| \| *Std. Dev.* \| \| *Corr* \| *Var* \| \| *Std. Dev.* \| \| *Corr* \| *Var* \| *Std. Dev.* \| *Corr* \| \| \| School.id \| Intercept  T2  T3 \| 0.34  0.22  0.27 \| 0.59  0.47  0.52 \| \| -.23 \| \| 0.34  0.23  0.34 \| 0.59  0.48  0.59 \| \| -.20 \| \| 0.37  0.46  0.36 \| 0.60  0.67  0.60 \| \| -.59 \| \| School \| Intercept  T2  T3 \| 0.002  -  - \| 0.05  -  - \| \|  \| \| 0.02  -  - \| 0.15  -  - \| \|  \| \| 0.21  0.18  0.11 \| 0.45  0.42  0.33 \| \|  \| \| Residual \|  \| 0.59 \| 0.77 \| \|  \| \| 0.54 \| 0.74 \| \|  \| \| 0.41 \| 0.64 \| \|  \| | \| Emotional Distress (H2) \| \| \| \| \| \| \| \| \| \| --- \| --- \| --- \| --- \| --- \| --- \| --- \| --- \| --- \| \| Depressive \| \| \| \| Anxious \| \| \| Stressed \| \| \| \| *Var* \| \| *Std.*  *Dev.* \| *Corr* \| *Var* \| *Std. Dev.* \| *Corr* \| *Var* \| *Std. Dev.* \| *Corr* \| \| 0.51  0.54  0.59  0.25  0.19  0.14  0.37 \| \| 0.71  0.73  0.77  0.50  0.44  0.37  0.61 \| -.51 \| 0.44  0.33  0.34  0.003  -  7.22 e^-9^  0.44 \| 0.67  0.57  0.58  0.06  -  8.50 e^-5^  0.66 \| -.27 \| 0.40  0.26  0.32  0.005  0.01  0.04  0.48 \| 0.63  0.51  0.56  0.07  0.12  0.21  0.69 \| -.24 \| |
| --- | --- | --- | --- | --- | --- | --- | --- | --- | --- | --- | --- | --- | --- | --- | --- | --- | --- | --- | --- | --- | --- | --- | --- | --- | --- | --- | --- | --- | --- | --- | --- | --- | --- | --- | --- | --- | --- | --- | --- | --- | --- | --- | --- | --- | --- | --- | --- | --- | --- | --- | --- | --- | --- | --- | --- | --- | --- | --- | --- | --- | --- | --- | --- | --- | --- | --- | --- | --- | --- | --- | --- | --- | --- | --- | --- | --- | --- | --- | --- | --- | --- | --- | --- | --- | --- | --- | --- | --- | --- | --- | --- | --- | --- | --- | --- | --- | --- | --- | --- | --- | --- | --- | --- | --- | --- | --- | --- | --- | --- | --- | --- | --- | --- | --- | --- | --- | --- | --- | --- | --- | --- | --- | --- | --- | --- | --- | --- | --- | --- | --- | --- | --- | --- | --- | --- | --- |

*Note. Var = variance, Std. Dev. = standard deviation, Corr = correlation*

**Table S3**

*Random Effects of Multilevel Models Estimating Putative Mediators (H1, H2, H3; ESM Data)*

|  |  | Dampening PA (H1) | | | | | Suppression NA (H2) | | | | | Non-Acceptance NA (H2) | | | | Pressure no NA (H3) | | |
| --- | --- | --- | --- | --- | --- | --- | --- | --- | --- | --- | --- | --- | --- | --- | --- | --- | --- | --- |
| Groups | Variable | *Var* | | *Std. Dev.* | | *Corr* | *Var* | | *Std. Dev.* | | *Corr* | *Var* | *Std. Dev.* | *Corr* | | *Var* | *Std. Dev.* | *Corr* |
| School.id | Intercept  T2  T3 | 0.54  0.42  0.40 | 0.74  0.65  0.63 | | -.43 | | 0.56  0.36  0.62 | 0.75  0.60  0.79 | | -.39 | | 0.50  0.47  0.48 | 0.71  0.69  0.69 | | -.48 | 0.44  0.27  0.52 | 0.66  0.52  0.72 | -.39 |
| School | Intercept  T2  T3 | 0.10  0.06  0.11 | 0.32  0.34  0.32 | |  | | 0.03  0.02  0.07 | 0.17  0.13  0.26 | |  | | 0.08  0.06  0.06 | 0.29  0.25  0.24 | |  | 0.08  0.05  0.16 | 0.29  0.23  0.40 |  |
| Residual |  | 0.32 | 0.56 | |  | | 0.39 | 0.62 | |  | | 0.37 | 0.61 | |  | 0.33 | 0.58 |  |

*Note. Var = variance, Std. Dev. = standard deviation, Corr = correlation*

**Table S4**

*Random Effects of Multilevel Models Estimating Main Outcomes (H1 and H2; SRQ Data)*

| \|  \|  \| Anhedonia (H1) \| \| \| \| --- \| --- \| --- \| --- \| --- \| \|  \|  \|  \|  \|  \| \| Groups \| Variable \| *Var* \| *Std.*  *Dev.* \| *Corr* \| \| School.id \| Intercept  T2  T3 \| 0.59  -  0.11 \| 0.77  -  0.33 \|  \| \| School \| Intercept  T2  T3  Condition (0)  Condition (1)  T3:Condition (0)  T3:Condition (1) \| -  -  -  0.04  0.10  -  - \| -  -  -  0.20  0.31  -  - \| -0.25 \| \| Residual \|  \| 0.31 \| 0.56 \|  \| | \| Emotional Distress (H2) \| \| \| \| \| \| \| \| \| \| \| \| --- \| --- \| --- \| --- \| --- \| --- \| --- \| --- \| --- \| --- \| --- \| \| Depressive \| \| \| \| \| Anxious \| \| \| \| Stressed \| \| \| \| *Var* \| \| *Std.*  *Dev.* \| *Corr* \| *Var* \| \| *Std. Dev.* \| *Corr* \| *Var* \| \| *Std. Dev.* \| *Corr* \| \| -  0.07  0.04  -  -  -  -  -  -  -  0.93 \| \| -  0.27  0.21  -  -  -  -  -  -  -  0.96 \|  \| 0.57  -  0.09  -  0.02  0.02  -  -  -  -  0.33 \| \| 0.75  -  0.29  -  0.13  0.14  -  -  -  -  0.57 \|  \| 0.49  -  0.12  -  0.02  0.02  -  -  0.01  0.07  0.35 \| \| 0.70  -  0.34  -  0.13  0.13  -  -  0.12  0.27  0.60 \| -.74 \| |
| --- | --- | --- | --- | --- | --- | --- | --- | --- | --- | --- | --- | --- | --- | --- | --- | --- | --- | --- | --- | --- | --- | --- | --- | --- | --- | --- | --- | --- | --- | --- | --- | --- | --- | --- | --- | --- | --- | --- | --- | --- | --- | --- | --- | --- | --- | --- | --- | --- | --- | --- | --- | --- | --- | --- | --- | --- | --- | --- | --- | --- | --- | --- | --- | --- | --- | --- | --- | --- | --- | --- | --- | --- | --- | --- | --- | --- | --- | --- |

*Note. Var = variance, Std. Dev. = standard deviation, Corr = correlation*

**Table S5**

*Random Effects of Multilevel Models Estimating Putative Mediators (H1, H2, H3; SRQ Data)*

|  |  | Dampening PA (H1) | | | | | Suppression NA (H2) | | | | | Non-Acceptance NA (H2) | | | | Pressure no NA (H3) | | |
| --- | --- | --- | --- | --- | --- | --- | --- | --- | --- | --- | --- | --- | --- | --- | --- | --- | --- | --- |
| Groups | Variable | *Var* | | *Std. Dev.* | | *Corr* | *Var* | | *Std. Dev.* | | *Corr* | *Var* | *Std. Dev.* | *Corr* | | *Var* | *Std. Dev.* | *Corr* |
| School.id | Intercept  T2  T3 | 0.72  -  - | 0.85  -  - | |  | | 0.56  -  0.19 | 0.75  -  0.43 | |  | | 0.55  -  0.04 | 0.74  -  0.19 | |  | 0.65  -  0.08 | 0.81  -  0.28 |  |
| School | Intercept  T2  T3 | -  -  - | -  -  - | |  | | -  -  - | -  -  - | |  | | -  -  - | -  -  - | |  | -  0.02  0.02 | -  0.15  0.16 |  |
| Residual |  | 0.26 | 0.51 | |  | | 0.36 | 0.60 | |  | | 0.40 | 0.64 | |  | 0.28 | 0.52 |  |

*Note. Var = variance, Std. Dev. = standard deviation, Corr = correlation*

**Post-Hoc Exploratory Analyses**

Multiple post-hoc exploratory analyses were conducted to rule out potential confounding or moderating factors (although this study was not powered for moderator analyses). First, a considerable number of participants had to be excluded from data analysis due to low ESM compliance (potentially compromising representativeness) even though SRQ data were available (details, see *Participant Flow Chart*). Post-hoc multilevel analyses were rerun **for all participants of whom SRQ data were available** for at least one assessment point irrespective of ESM compliance (see *Descriptive Statistics* in Table S6).

**Table S6**

*Descriptive Statistics for Raw Outcome Scores at Each Assessment Point (All SRQ Data)*

| Variable | T1  *M (SD)* | | *SMD* | T2  *M (SD)* | | T3  *M (SD)* | |
| --- | --- | --- | --- | --- | --- | --- | --- |
|  | Control | MT |  | Control | MT | Control | MT |
| **SRQ measures** |  |  |  |  |  |  |  |
| Depressive symptoms  (DASS-D) | 10.05 (8.45) | 9.93 (8.65) | 0.014 | 10.34 (9.06) | 8.81 (8.52) | 10.08 (8.03) | 9.54 (9.54) |
| Anxious symptoms  (DASS-A) | 9.57 (7.84) | 9.19 (7.67) | 0.049 | 10.83 (7.97) | 10.31 (8.95) | 10.19 (8.08) | 10.18 (9.86) |
| Stress  (DASS-S) | 12.29 (8.52) | 12.01 (8.04) | 0.033 | 12.57 (8.60) | 13.23 (9.08) | 12.48 (9.08) | 12.93 (10.33) |
| Anhedonia  (LASS) | 23.75 (9.91) | 22.85 (9.35) | 0.093 | 23.67 (10.32) | 22.67 (9.72) | 24.45 (10.04) | 23.13 (10.79) |
| Non-Acceptance NA  (NASNES-NA) | 18.80 (7.30) | 18.98 (7.29) | 0.025 | 17.41 (7.12) | 17.02 (7.55) | 17.84 (7.11) | 16.14 (7.77) |
| Suppression NA  (NASNES-S) | 22.77 (6.97) | 22.78 (7.34) | 0.001 | 21.33 (7.31) | 20.14 (7.60) | 21.04 (7.37) | 18.95 (8.34) |
| Dampening PA  (RPA-D) | 11.09 (3.80) | 10.68 (3.64) | 0.110* | 10.33 (3.52) | 10.29 (3.82) | 10.27 (3.41) | 10.26 (4.17) |
| Perceived social pressure non-experience/expression of NA (SEDAS-NEG) | 55.86 (15.53) | 55.21 (17.37) | 0.039 | 53.27 (17.03) | 52.26 (17.02) | 51.72 (18.09) | 49.34 (17.14) |
|  |  |  |  |  |  |  |  |

*Note.* *N* _control_ = 231; *N* _intervention_ = 221; T1 = baseline, T2 = post-intervention, T3 = follow-up ; NA = Negative Affect/emotions, PA = Positive Affect/emotions ; SMD = Standardized Mean Difference, > .10 are indicated by an *.

As presented in Table S7 and S8 (see also Figures S5 and S6, and Table S9 and S10 for random effects), estimates for the fixed effects were largely in line with the SRQ data of the participants of whom both ESM and SRQ data were available. For depressive symptoms at post-training (Estimate _T2:Condition_ = -2.12, *p* *_uncorrected_* = .046, *p* *_corrected_* = .37) and for suppression of negative emotions at follow-up (Estimate _T3:Condition_ = -2.50, *p* *_uncorrected_* = .01, *p* _corrected_ = .08), significant time x condition interaction effects were found. However, these effects became insignificant after correction for multiple testing. Bootstrapping results, not corrected for potential bias, largely confirmed these trends (Table S11 and S12), showing a significant decrease in depressive symptoms for the MT group at post-training (Estimate _T2:Condition_ = -0.20, 95% CI [-0.37; -0.05]), and in suppression and non-acceptance of NA at follow-up (Estimate _T3:Condition_ = -0.24, 95% CI [-0.41; -0.02] and Estimate _T3:Condition_ = -0.21, 95% CI [-0.42; -0.009]). Taken together, given effects did not withstand correction for multiple testing, no strong support was found for a substantial lasting impact of universal school-based MT in this larger dataset either. The presence of decreasing tendencies in the data though might again point towards the potentially crucial role an interplay of moderating factors.

**Table S7**

*Main and Interaction Fixed Effects of Multilevel Model Estimating Main Outcomes (H1, H2; All SRQ Data)*

|  | Anhedonia (H1) | | | | Emotional Distress (H2) | | | | | | | | | | |
| --- | --- | --- | --- | --- | --- | --- | --- | --- | --- | --- | --- | --- | --- | --- | --- |
|  |  | | | | Depressive | | | Anxious | | | | | Stressed | | |
|  | | Est.  (SE) | | *t(p)*  *df* | Est.  (SE) | | *t(p)*  *df* | | Est.  (SE) | | *t(p)*  *df* | Est.  (SE) | | | *t(p*  *df)* |
| Intercept | | -0.12  (0.11) | -1.10 (.29)  17.41 | | -0.17  (0.10) | -1.65 (.12)  18.79 | | -0.39  (0.08) | | -4.71 (<.001)**  550.76 | | -0.42  (0.08) | | -5.01 (<.001)**  19.94 | |
| Age | | 0.01  (0.06) | 0.20 (.84)  54.51 | | -0.003  (0.06) | -0.06 (.95)  61.32 | | -0.03  (0.04) | | -0.67 (.50)  460.37 | | 0.04  (0.05) | | 0.83 (.41)  25.15 | |
| Gender | | 0.22  (0.09) | 2.61 (.01)*  435.38 | | 0.33  (0.08) | 3.94 (<.001)**  439.30 | | 0.51  (0.08) | | 6.22 (<.001)**  435.28 | | 0.58  (0.08) | | 7.34 (<.001)**  426.56 | |
| T2 | | 0.02  (0.06) | 0.41 (.68)  487.55 | | 0.05  (0.06) | 0.84 (.40)  128.56 | | 0.19  (0.10) | | 1.89 (.09)  10.98 | | 0.06  (0.07) | | 0.95 (.35)  20.98 | |
| T3 | | 0.09  (0.06) | 1.53 (.13)  607.00 | | 0.03  (0.07) | 0.40 (.69)  35.12 | | 0.10  (0.09) | | 1.10 (.29)  15.39 | | 0.03  (0.07) | | 0.52 (.60)  580.67 | |
| Condition | | -0.08  (0.13) | -0.63 (.54)  11.98 | | -0.02  (0.12) | -0.15 (.88)  13.16 | | -0.03  (0.09) | | -0.35 (.72)  630.40 | | -0.02  (0.10) | | -0.16 (.89)  15.18 | |
| T2:Condition | | -0.07  (0.08) | -0.90 (.37)  486.85 | | -0.20  (0.10) | -2.12 (.046*/.37)⸷  21.00 | | -0.09  (0.12) | | -0.74 (.47)  13.73 | | 0.07  (0.09) | | 0.81 (.42)  485.59 | |
| T3:Condition | | -0.08  (0.09) | -0.88 (.38)  601.53 | | -0.10  (0.09) | -1.07 (.29)  634.91 | | 0.02  (0.09) | | 0.23 (.82)  579.20 | | 0.06  (0.10) | | 0.65 (.52)  575.09 | |

*Note*. * *p* < .05; ** *p* < .001 ; ⸷ Effect no longer significant after Benjamini-Hochberg (BH) correction for multiple testing, respectively uncorrected and BH-corrected *p*-value between brackets.

**Table S8**

*Main and Interaction Fixed Effects of Multilevel Model Estimating Outcomes Putative Mediators (H1, H2, H3; All SRQ Data)*

|  | Dampening (H1) | | | Suppression NA (H2) | | Non-Acceptance NA (H2) | | Pressure non-expression/experience of NA (H3) | | |
| --- | --- | --- | --- | --- | --- | --- | --- | --- | --- | --- |
|  | Est.  (SE) | | *t (p)*  *df* | Est.  (SE) | *t(p)*  *df* | Est.  (SE) | *t(p)*  *df* | Est.  (SE) | *t(p)*  *df* | |
| Intercept | -0.04  (0.11) | -0.38 (.71)  18.36 | | 0.07  (0.08) | 0.84 (.40)  577.38 | -0.16  (0.08) | -1.97 (.049)*  597.75 | 0.12  (0.10) | | 1.12 (.27)  27.66 |
| Age | 0.05  (0.06) | 0.89 (.37)  78.33 | | -0.07  (0.04) | -1.69 (.09)  411.17 | 0.008  (0.04) | 0.18 (.86)  435.77 | 0.0007  (0.06) | 0.01 (.99)  87.25 | |
| Gender | 0.34  (0.09) | 3.99 (<.001)**  432.55 | | 0.20  (0.08) | 2.46 (.01)*  438.42 | 0.46  (0.08) | 5.63 (<.001)**  441.43 | 0.05  (0.09) | 0.54 (.59)  429.90 | |
| T2 | -0.18  (0.07) | -2.80 (.01)*  23.63 | | -0.19  (0.07) | -2.82 (.008)*  28.46 | -0.18  (0.06) | -2.89 (.004)*  482.71 | -0.18  (0.07) | -2.73 (.01)*  16.05 | |
| T3 | -0.19  (0.05) | -3.59 (<.001)**  782.52 | | -0.25  (0.07) | -3.85 (<.001)**  609.17 | -0.17  (0.08) | -2.22 (.04)*  13.98 | -0.26  (0.07) | -3.78 (.001)*  21.05 | |
| Condition | -0.11  (0.11) | -0.96 (.36)  11.19 | | -0.002  (0.09) | -0.02 (.99)  667.71 | 0.04  (0.09) | 0.44 (.66)  694.66 | -0.02  (0.12) | -0.15 (.88)  9.77 | |
| T2:Condition | 0.09  (0.08) | 1.13 (.26)  791.30 | | -0.13  (0.09) | -1.39 (.16)  486.29 | -0.08  (0.09) | -0.84 (.40)  482.26 | 0.03  (0.08) | 0.41 (.69)  437.70 | |
| T3:Condition | 0.06  (0.09) | 0.79 (.43)  784.80 | | -0.24  (0.09) | -2.50 (.01*/.08)⸷  604.98 | -0.21  (0.12) | -1.67 (.12)  11.08 | -0.05  (0.09) | -0.61 (.54)  579.60 | |

*Note*. * *p* < .05; ** *p* < .001 ; ⸷ Effect no longer significant after Benjamini-Hochberg (BH) correction for multiple testing, respectively uncorrected and BH-corrected *p*-value between brackets.

**Figure S5**

*Main and Interaction Fixed Effects of Multilevel Model Estimating Main Outcomes (H1, H2; All SRQ Data)*


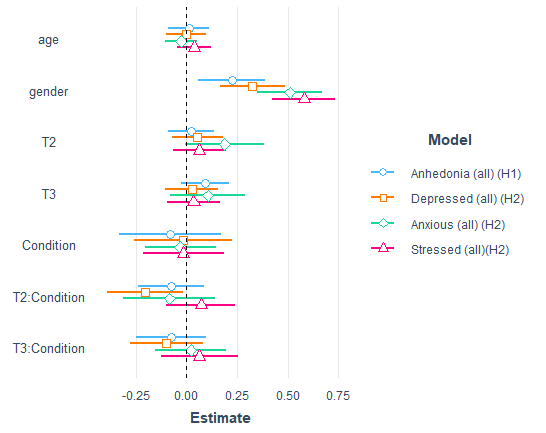


*Note.* If the 95% CI includes zero, then then estimate is considered as insignificant.

**Figure S6**

*Main and Interaction Fixed Effects of Multilevel Model Estimating Putative Mediators (H1, H2, H3; All SRQ Data)*


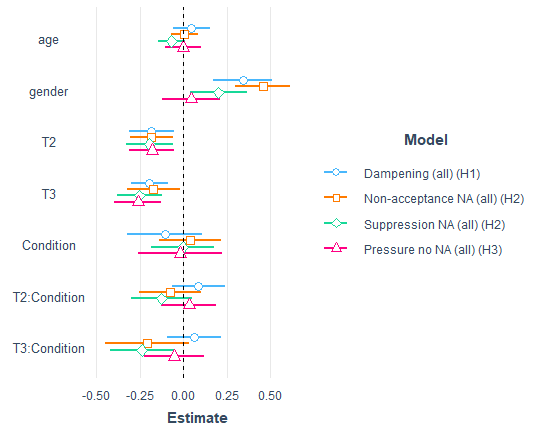


*Note.* If the 95% CI includes zero, then then estimate is considered as insignificant.

**Random Effects of Multilevel Models Estimating Main Outcomes and Putative Mediators (All SRQ Data)**

**Table S9**

*Random Effects of Multilevel Models Estimating Main Outcomes (H1 and H2; All SRQ Data)*

| \|  \|  \| Anhedonia (H1) \| \| \| \| --- \| --- \| --- \| --- \| --- \| \|  \|  \|  \|  \|  \| \| Groups \| Variable \| *Var* \| *Std.*  *Dev.* \| *Corr* \| \| School.id \| Intercept  T2  T3 \| 0.57  -  0.07 \| 0.75  -  0.26 \|  \| \| School \| Intercept  T2  T3  Condition (0)  Condition (1)  T2:Condition (0)  T2:Condition (1)  T3:Condition (0)  T3:Condition (1) \| -  -  -  0.05  0.06  -  -  -  - \| -  -  -  0.22  0.24  -  -  -  - \| .21 \| \| Residual \|  \| 0.35 \| 0.59 \|  \| | \| Emotional Distress (H2) \| \| \| \| \| \| \| \| \| \| \| \| --- \| --- \| --- \| --- \| --- \| --- \| --- \| --- \| --- \| --- \| --- \| \| Depressive \| \| \| \| \| Anxious \| \| \| \| Stressed \| \| \| \| *Var* \| \| *Std.*  *Dev.* \| *Corr* \| *Var* \| \| *Std. Dev.* \| *Corr* \| *Var* \| \| *Std. Dev.* \| *Corr* \| \| 0.50  -  0.03  -  -  0.003  0.04  0.07  0.0007  0.005  -  -  0.42 \| \| 0.71  -  0.16  -  -  0.060.20  0.26  0.03  0.07  -  -  0.65 \| .35  -1.00 \| 0.53  -  0.08  -  -  0.05  -  -  0.07  0.02  -  -  0.35 \| \| 0.73  -  0.28  -  -  0.23  -  -  0.26  0.15  -  -  0.59 \| .32 \| 0.45  -  0.17  -  0.007  -  0.006  0.034  -  -  -  -  0.38 \| \| 0.67  -  0.41  -  0.08  -  0.08  0.18  -  -  -  -  0.62 \| .47 \| |
| --- | --- | --- | --- | --- | --- | --- | --- | --- | --- | --- | --- | --- | --- | --- | --- | --- | --- | --- | --- | --- | --- | --- | --- | --- | --- | --- | --- | --- | --- | --- | --- | --- | --- | --- | --- | --- | --- | --- | --- | --- | --- | --- | --- | --- | --- | --- | --- | --- | --- | --- | --- | --- | --- | --- | --- | --- | --- | --- | --- | --- | --- | --- | --- | --- | --- | --- | --- | --- | --- | --- | --- | --- | --- | --- | --- | --- | --- | --- |

*Note. Var = variance, Std. Dev. = standard deviation, Corr = correlation*

**Table S10**

*Random Effects of Multilevel Models Estimating Putative Mediators (H1, H2, H3; All SRQ Data)*

|  |  | Dampening PA (H1) | | | | | Suppression NA (H2) | | | | | Non-Acceptance NA (H2) | | | | Pressure no NA (H3) | | |
| --- | --- | --- | --- | --- | --- | --- | --- | --- | --- | --- | --- | --- | --- | --- | --- | --- | --- | --- |
| Groups | Variable | *Var* | | *Std. Dev.* | | *Corr* | *Var* | | *Std. Dev.* | | *Corr* | *Var* | *Std. Dev.* | *Corr* | | *Var* | *Std. Dev.* | *Corr* |
| School.id | Intercept  T2  T3 | 0.60  -  - | 0.78  -  - | |  | | 0.52  -  0.08 | 0.72  -  0.28 | |  | | 0.50  -  0.01 | 0.71  -  0.12 | |  | 0.57  -  0.13 | 0.75  -  0.36 |  |
| School | Intercept  T2  T3  Condition (0)  Condition (1)  T2:Condition (0)  T2:Condition (1)  T3:Condition (0)  T3:Condition (1) | -  0.01  -  0.05  0.06  -  -  -  - | -  0.11  -  0.22  0.24  -  -  -  - | | .65 | | -  0.008  -  -  -  -  -  -  - | -  0.09  -  -  -  -  -  -  - | |  | | -  -  -  -  -  -  -  0.02  0.01 | -  -  -  -  -  -  -  0.14  0.12 | | -1.00 | -  0.01  0.01  0.04  0.09  -  -  -  - | -  0.11  0.10  0.19  0.30  -  -  -  - | .47 |
| Residual |  | 0.31 | 0.56 | |  | | 0.41 | 0.64 | |  | | 0.41 |  | |  | 0.32 | 0.56 |  |
|  |  |  |  | |  | |  |  | |  | |  |  | |  |  |  |  |

*Note. Var = variance, Std. Dev. = standard deviation, Corr = correlation*

**Main and Interaction Fixed Effects of Multilevel Model Estimating Outcomes for all SRQ Data Based on Wild Bootstrapping Technique**

Table S11

*Main and Interaction Fixed Effects of Multilevel Model Estimating Main Outcomes (H1, H2; All SRQ Data) With Bootstrapping*

|  | | Anhedonia (H1) | | | | Emotional Distress (H2) | | | | | | | | | | | |  |
| --- | --- | --- | --- | --- | --- | --- | --- | --- | --- | --- | --- | --- | --- | --- | --- | --- | --- | --- |
|  | |  | | | | Depressive | | | | Anxious | | | | Stressed | | | | |
|  | | Est. | | 95% CI | Est. | | 95% CI | | | Est. | | 95% CI | | Est. | | 95% CI |  |  |
| Intercept | | -0.12 | [-0.28; 0.12] | | -0.17 | [-0.33; -0.03]* | | -0.39 | | | [-0.50; -0.23]* | | | -0.42 | [-0.57; -0.29]* | | |  |
| Age | | 0.01 | [-0.09; 0.10] | | -0.003 | [-0.09; 0.09] | | -0.03 | | | [-0.10; 0.06] | | | 0.04 | [-0.04; 0.10] | | |  |
| Gender | | 0.22 | [0.003; 0.39]* | | 0.33 | [0.17; 0.46]* | | 0.51 | | | [0.35; 0.66]* | | | 0.58 | [0.44; 0.68]* | | |  |
| T2 | | 0.02 | [-0.08; 0.16] | | 0.05 | [-0.06; 0.18] | | 0.19 | | | [0.04; 0.35]* | | | 0.06 | [-0.06; 0.17] | | |  |
| T3 | | 0.09 | [-0.02; 0.24] | | 0.03 | [-0.09; 0.15] | | 0.10 | | | [-0.04; 0.24] | | | 0.03 | [-0.12; 0.16] | | |  |
| Condition | | -0.08 | [-0.27; 0.09] | | -0.02 | [-0.18; 0.18] | | -0.03 | | | [-0.20; 0.14] | | | -0.02 | [-0.16; 0.17] | | |  |
| T2:Condition | | -0.07 | [-0.21; 0.09] | | -0.20 | [-0.37; -0.05]* | | -0.09 | | | [-0.28; 0.07] | | | 0.07 | [-0.11; 0.21] | | |  |
| T3:Condition | | -0.08 | [-0.24; 0.06] | | -0.10 | [-0.27; 0.08] | | 0.02 | | | [-0.18; 0.19] | | | 0.06 | [-0.11; 0.25] | | |  |

*Note.* If zero is not included in the 95% CI the estimate can be considered as significant (*).

**Table S12**

*Main and Interaction Fixed Effects of Multilevel Model Estimating Outcomes Putative Mediators (H1, H2, H3; All SRQ Data) With Bootstrapping*

|  | Dampening (H1) | | | Suppression NA (H2) | | Non-Acceptance NA (H2) | | Pressure non-expression/experience of NA (H3) | |
| --- | --- | --- | --- | --- | --- | --- | --- | --- | --- |
|  | Est. | 95% CI | | Est. | 95% CI | Est. | 95% CI | Est. | 95% CI |
| Intercept | -0.04 | | [-0.23; 0.17] | 0.07 | [-0.09; 0.23] | -0.16 | [-0.31; 0.04] | 0.12 | [-0.01; 0.25] |
| Age | 0.05 | | [-0.02; 0.13] | -0.07 | [-0.16; 0.02] | 0.008 | [-0.07; 0.08] | 0.001 | [-0.06; 0.07] |
| Gender | 0.34 | | [0.16; 0.50]* | 0.20 | [0.05; 0.34]* | 0.46 | [0.32; 0.64]* | 0.05 | [-0.07; 0.18] |
| T2 | -0.18 | | [-0.27; -0.09]* | -0.19 | [-0.32; -0.08]* | -0.18 | [-0.33; -0.07]* | -0.18 | [-0.27; -0.09]* |
| T3 | -0.20 | | [-0.20; -0.27]* | -0.25 | [-0.39; -0.10]* | -0.17 | [-0.33; -0.05]* | -0.26 | [-0.37; -0.14]* |
| Condition | -0.11 | | [-0.33; 0.06] | -0.002 | [-0.14; 0.18] | 0.04 | [-0.15; 0.23] | -0.02 | [-0.18; 0.18] |
| T2:Condition | 0.09 | | [-0.06; 0.28] | -0.13 | [-0.31; 0.03] | -0.08 | [-0.23; 0.13] | 0.03 | [-0.12; 0.17] |
| T3:Condition | 0.06 | | [-0.23; 0.23] | -0.24 | [-0.41; -0.02]* | -0.21 | [-0.42; -0.009]* | -0.05 | [-0.26; 0.18] |

*Note.* If zero is not included in the 95% CI the estimate can be considered as significant (*).

Second, the potential moderating role of **levels of ESM compliance** was explored, because individuals’ mean ESM compliance could be considered as an indicator of the extent of engagement in the assessment phases. As presented in Table S13, for each assessment point, considerable between-person variability was found concerning ESM compliance rates. Consequently, individuals’ overall level of ESM compliance was added to the multilevel models as a moderator (i.e., two-way interaction effects with time/condition, and three-way interaction effects with time and condition). However, analyses on the ESM and SRQ data did not reveal significant interactions implying that findings depend on individuals’ level of ESM compliance.

**Table S13**

*Descriptive Statistics for ESM Compliance Grouped by Assessment Point and Condition*

|  | Control | | | | Intervention | | | | |
| --- | --- | --- | --- | --- | --- | --- | --- | --- | --- |
| % compliance | *M* | *SD* | Min | Max | | *M* | *SD* | Min | Max |
| T1 | 72.12 | 16.63 | 27.50 | 100 | | 69.82 | 18.37 | 20.00 | 97.50 |
| T2 | 57.72 | 18.39 | 20.00 | 97.5 | | 60.96 | 20.34 | 20.00 | 92.50 |
| T3 | 56.16 | 19.60 | 20.00 | 100 | | 57.95 | 21.32 | 20.00 | 95.00 |

Third, **baseline level depressive symptomatology**, measured via the SRQs, was added as a moderator to the model predicting depressive symptoms and anhedonia at post-training and follow-up (i.e., three-way interaction effects with time and condition). In other words, it was explored whether decreases in symptomatology at post-training and follow-up were more pronounced for adolescents with higher levels of baseline depressive symptomatology. Given the increase in model complexity and this study not being powered for moderation analyses, analyses were run on all SRQ data available irrespective of ESM compliance and gender was treated as a binary (i.e., female/male) variable (i.e., excluding 4 and 2 participants from the control and MT group respectively; *n* _control_ = 227 and *n* _intervention_ = 219). For depressive symptoms, no significant three-way interactions were found either at post-training or follow-up (Estimate _T2:Condition:BaselineDASS_D_ = -0.02, *p* = .81; Estimate _T3:Condition:BaselineDASS_D_ = 0.09, *p* = .31). Similarly, no moderating effect of baseline depressive symptomatology was found for anhedonia at post-training or follow-up (Estimate _T2:Condition:BaselineDASS_D_ = 0.04, *p* = .65; Estimate _T3:Condition:BaselineDASS_D_ = 0.15, *p* = .08).

Fourth, the role that **engagement with the MT** plays in symptom change, reflected in reported levels of home practice, was explored by running multilevel models on all data available of participants in the intervention group (*n* = 219). In these models, frequency of formal home practice (FHP) was included as predictor for outcomes at post-training and follow-up, on top of age and gender as covariates. Due to similar convergence issues as for the primary analyses, model comparison was conducted per outcome variable (see procedure Data Analyses). As presented in Table S14 and S15, higher levels of formal home practice during the course of the MT (i.e., reported at post-training assessment) predict lower levels of anhedonia at post-training (Estimate _T2:FHP_ = -0.10, *p* _uncorrected_ = .004, *p* _corrected_ = .03). At follow-up, this effect was not significant after correcting for multiple testing (Estimate _T3:FHP_ = -0.10, *p* _uncorrected_ = .008, *p* _corrected_ = .06). However, models including reported levels of home practice during the follow-up period (i.e., reported at follow-up assessment) show a slightly different data pattern (Table S16 and S17). Here, in contrast to our expectations, positive associations between FHP and depressive symptoms at follow-up (Estimate _T3:FHP_ = 0.10, *p* _uncorrected_ = .01, *p* _corrected_ = .08) and non-acceptance of negative emotions (Estimate _T3:FHP_ = 0.08, *p* _uncorrected_ = .03, *p* _corrected_ = .12) were found, albeit not statistically significant after multiple testing correction. Taken together, these findings lend some preliminary support for the role of engagement with the MT to initiate symptom improvement. However, given the absence of consistent strong effects, future research is needed which ideally maps engagement with the MT in an even more systematic and fine-grained way (e.g., daily reporting of level of home practice, types of exercises most frequently practiced with).

**Table S14**

*Main and Interaction Fixed Effects of Multilevel Model Estimating Main Outcomes (H1, H2), Role Frequency of Home Practice (Formal, During MT) (All SRQ Data)*

|  | Anhedonia (H1) | | | | Emotional Distress (H2) | | | | | | | | | | | | |
| --- | --- | --- | --- | --- | --- | --- | --- | --- | --- | --- | --- | --- | --- | --- | --- | --- | --- |
|  |  | | | | Depressive | | | | Anxious | | | | | | Stressed | | |
|  | | Est.  (SE) | | *t(p)*  *df* | Est.  (SE) | | *t(p)*  *df* | | | Est.  (SE) | | *t(p)*  *df* | | Est.  (SE) | | | *t(p)*  *df* |
| Intercept | | -0.25 (0.13) | -1.98 (.0495)*  207.01 | | -0.21 (0.16) | -1.28 (.22)  16.57 | | -0.48  (0.12) | | | -3.94 (.0001)**  211.32 | | -0.49  (0.11) | | | -4.27 (2.85e^-05^)**  229.16 | |
| Age | | -0.06  (0.08) | -0.69 (.49)  173.36 | | 0.10 (0.07) | 1.31 (.19)  166.42 | | -0.02 (0.08) | | | -0.25 (.80)  102.55 | | 0.02  (0.07) | | | 0.33 (.74)  99.40 | |
| Gender | | 0.32  (0.13) | 2.36 (.020)*  166.92 | | 0.31  ( 0.09) | 3.38 (.0008)**  504.94 | | 0.59 (0.13) | | | 4.58 (8.8e^-06^) **  172.33 | | 0.73  (0.12) | | | 6.07 (7.75e^-09^)**  173.70 | |
| T2 | | 0.09  (0.08) | 1.12 (.26)  222.16 | | -0.17 (0.13) | -1.26 (.21)  339.06 | | 0.14  (0.08) | | | 1.90 (.06)  204.90 | | 0.11  (0.08) | | | 1.25 (.21)  220.08 | |
| T3 | | 0.19  (0.10) | 1.85 (.08)  21.14 | | -0.10 (0.15) | -0.68 (.50)  369.88 | | 0.22  (0.11) | | | 1.93 (.07)  17.11 | | 0.14  (0.13) | | | 1.09 (.29)  16.07 | |
| FHP | | 0.07  (0.08) | 0.89 (.40)  8.79 | | 0.06 (0.08) | 0.06 (.08)  10.00 | | 0.04  (0.05) | | | 0.80 (.45)  7.72 | | -0.01 (0.05) | | | -0.27 (.80)  14.68 | |
| T2:FHP | | -0.10  (0.03) | -2.92 (.004*/.03*)  222.16 | | -0.01 (0.05) | -0.18 (.86)  339.06 | | -0.04  (0.03) | | | -1.35 (.18)  204.90 | | 0.01  (0.04) | | | 0.21 (.83)  220.08 | |
| T3:FHP | | -0.10  (0.04) | -2.65 (.008*/.06)⸷  240.93 | | 0.01 (0.06) | 0.09 (.93)  374.59 | | -0.07  (0.04) | | | -1.86 (.06)  231.12 | | -0.02 (0.04) | | | -0.42 (.68)  230.13 | |

*Note*. * *p* < .05; ** *p* < .001; intervention group only (‘all data’, irrespective of ESM compliance; *n*  = 219); FHP = Frequency of Home Practice; In case of significant uncorrected *p*-values, corrected *p*-values are added between brackets. ⸷ Uncorrected effect becomes insignificant after correction for multiple testing following Benjamini-Hochberg procedure (1995).

**Table S15**

*Main and Interaction Fixed Effects of Multilevel Model Estimating Outcomes Putative Mediators (H1, H2, H3) - Role Frequency of Home Practice (Formal, During MT) (All SRQ Data)*

|  | Dampening (H1) | | | Suppression NA (H2) | | Non-Acceptance NA (H2) | | Pressure non-expression/experience  of NA (H3) | | | |  |
| --- | --- | --- | --- | --- | --- | --- | --- | --- | --- | --- | --- | --- |
|  | Est.  (SE) | | *t (p)*  *df* | Est.  (SE) | *t(p)*  *df* | Est.  (SE) | *t(p)*  *df* | Est.  (SE) | | *t(p)*  *df* | |  |
| Intercept | -0.28  (0.13) | -2.15 (.03)*  215.01 | | 0.12  (0.14) | 0.89 (.39)  22.45 | -0.13  (0.13) | -0.97 (.34)  33.75 | | 0.13  (0.16) | | 0.85 (.41)  18.81 | |
| Age | 0.02  (0.08) | 0.29 (.78)  83.68 | | -0.31  (0.07) | -4.64 (<.001)**  69.31 | -0.24  (0.06) | -3.65 (<.001)**  82.47 | 0.005  (0.09) | | 0.05 (.96)  58.40 | |  |
| Gender | 0.45  (0.14) | 3.31 (.001)*  175.25 | | 0.25  (0.09) | 2.87 (.004)*  502.07 | 0.55  (0.09) | 6.39 (<.001)**  514.63 | -0.01  (0.13) | | -0.11 (.92)  173.59 | |  |
| T2 | -0.01  (0.08) | -0.13 (.90)  212.24 | | -0.21  (0.13) | -1.63 (.11)  27.85 | -0.26  (0.13) | -2.01 (.04)*  350.36 | -0.11  (0.08) | | -1.29 (.20)  184.11 | |  |
| T3 | -0.01  (0.09) | -0.14 (.89)  248.48 | | -0.42  (0.14) | -2.97 (.003)*  382.64 | -0.42  (0.14) | -3.03 (.003)*  399.75 | -0.30  (0.10) | | -2.98 (.003)**  246.21 | |  |
| FHP | 0.04  (0.05) | 0.81 (.43)  12.59 | | -0.03  (0.04) | -0.66 (.51)  360.40 | -0.01  (0.04) | -0.25 (.80)  361.69 | 0.04  (0.05) | | 0.87 (.40)  12.99 | |  |
| T2: FHP | -0.05  (0.03) | -1.56 (.12)  212.24 | | -0.06  (0.05) | -1.08 (.28)  296.97 | -0.004  (0.05) | -0.07 (.95)  350.35 | -0.04  (0.04) | | -0.97 (.35)  20.52 | |  |
| T3: FHP | -0.07  (0.04) | -1.82 (.07)  250.48 | | -0.03  (0.06) | -0.66 (.51)  386.00 | 0.02  (0.06) | 0.29 (.78)  403.54 | -0.02  (0.04) | | -0.53 (.60)  248.50 | |  |

*Note*. * *p* < .05; ** *p* < .001; intervention group only (“all data’, irrespective of ESM compliance; *n* = 219); FHP = Frequency of Home Practice

**Table S16**

*Main and Interaction Fixed Effects of Multilevel Model Estimating Main Outcomes (H1, H2), Role Frequency of Home Practice (Formal, During Follow-Up) (All SRQ Data)*

|  | Anhedonia (H1) | | Emotional Distress (H2) | | | | | | | | | |
| --- | --- | --- | --- | --- | --- | --- | --- | --- | --- | --- | --- | --- |
|  |  | | Depressive | | | Anxious | | | Stressed | | | |
|  | Est.  (SE) | *t(p)*  *df* | | Est.  (SE) | *t(p)*  *df* | | Est.  (SE) | *t(p)*  *df* | | Est.  (SE) | *t(p)*  *df* |  |
| Intercept | -0.15  (0.13) | -1.12 (.27)  33.68 | | -0.14  (0.13) | -1.08 (.29)  34.47 | | -0.44  (0.14) | -3.26 (.003)*  22.62 | | -0.52  (0.11) | -4.63 (<.001)**  29.22 |  |
| Age | 0.03  (0.09) | 0.30 (.77)  53.54 | | 0.08  (0.08) | 0.94 (.35)  53.20 | | 0.03  (0.08) | 0.35 (.73)  72.05 | | 0.10  (0.07) | 1.32 (.19)  38.75 |  |
| Gender | 0.21  (0.12) | 1.72 (.09)  207.10 | | 0.31  (0.12) | 2.61 (.010)*  211.80 | | 0.47  (0.11) | 4.07 (<.001)**  209.00 | | 0.63  (0.11) | 5.67 (<.001)**  205.70 |  |
| T2 | -0.05  (0.06) | -0.93 (.35)  246.10 | | -0.15  (0.06) | -2.40 (.02)*  242.10 | | 0.10  (0.06) | 1.69 (.13)  7.52 | | 0.14  (0.06) | 2.12 (.09)  5.14 |  |
| T3 | 0.02  (0.08) | 0.28 (.78)  17.22 | | -0.18  (0.09) | -2.12 (.049)*  16.75 | | 0.05  (0.09) | 0.54 (.60)  13.54 | | 0.08  (0.10) | 0.76 (.47)  9.15 |  |
| FHP | -0.00004  (0.0002) | -0.24 (.81)  231.70 | | -0.0002  (0.0002) | -0.66 (.51)  246.30 | | 0.0003  (0.0002) | 1.60 (.11)  243.70 | | 0.0003  (0.0002) | 1.72 (.09)  222.60 |  |
| T3: FHP | -0.003  (0.04) | -0.09 (.93)  177.50 | | 0.10  (0.04) | 2.52 (.01*/.08)⸷  179.00 | | 0.05  (0.04) | 1.45 (.15)  177.70 | | 0.03  (0.04) | 0.69 (.49)  176.10 |  |

*Note*. * *p* < .05; ** *p* < .001; intervention group only (“all data’, irrespective of ESM compliance; *n* = 219); FHP = Frequency of Home Practice; In case of significant uncorrected *p*-values, corrected *p*-values are added between brackets. ⸷ Uncorrected effect becomes insignificant after correction for multiple testing following Benjamini-Hochberg procedure (1995).

**Table S17**

*Main and Interaction Fixed Effects of Multilevel Model Estimating Outcomes Putative Mediators (H1, H2, H3) - Role Frequency of Home Practice (Formal, During Follow-Up) (All SRQ Data)*

|  | Dampening (H1) | | | Suppression NA (H2) | | Non-Acceptance NA (H2) | | Pressure non-expression/experience  of NA (H3) | | | |
| --- | --- | --- | --- | --- | --- | --- | --- | --- | --- | --- | --- |
|  | Est.  (SE) | | *t (p)*  *df* | Est.  (SE) | *t(p)*  *df* | Est.  (SE) | *t(p)*  *df* | Est.  (SE) | *t(p)*  *df* | | |
| Intercept | -0.15  (0.13) | -1.22 (.23)  37.09 | | 0.04  (0.11) | 0.39 (.70)  37.79 | -0.17  (0.11) | -1.53 (.13)  50.24 | 0.14  (0.14) | | 0.96 (.35)  20.23 |  |
| Age | 0.06  (0.08) | 0.79 (.43)  50.71 | | -0.19  (0.07) | -2.67 (.01)*  19.99 | -0.12  (0.07) | -1.59 (.12)  39.53 | -0.008  (0.09) | -0.10 (.92)  62.90 | | |
| Gender | 0.33  (0.12) | 2.74 (.007)*  208.20 | | 0.26  (0.11) | 2.31 (.02)*  210.40 | 0.53  (0.12) | 4.62 (<.001)**  211.50 | 0.02  (0.12) | 0.15 (.88)  206.50 | | |
| T2 | -0.08  (0.07) | -1.06 (.32)  8.679 | | -0.31  (0.08) | -3.96 (.002)*  12.18 | -0.25  (0.06) | -4.09 (<.001)**  237.80 | -0.14  (0.07) | -2.02 (.06)  13.74 | | |
| T3 | -0.17  (0.07) | -2.33 (.02)*  238.40 | | -0.49  (0.09) | -5.77 (<.001)**  14.68 | -0.47  (0.08) | -6.17 (<.001)**  260.70 | -0.38  (0.08) | -4.66 (<.001)**  12.53 | | |
| FHP | 0.00007  (0.0002) | 0.38 (.71)  232.70 | | 0.0002  (0.0002) | 1.37 (.17)  190.30 | 0.0001  (0.0002) | 0.69 (.49)  236.00 | 0.0001  (0.0002) | 0.81 (.42)  254.40 | | |
| T3: FHP | 0.04  (0.03) | 1.13 (.26)  176.40 | | 0.04  (0.04) | 0.88 (.38)  178.30 | 0.08  (0.04) | 2.23 **(.03***/.12)⸷  179.10 | 0.07  (0.04) | 1.79 (.08)  177.70 | | |

*Note*. * *p* < .05; ** *p* < .001; intervention group only (“all data’, irrespective of ESM compliance; *n* = 219); FHP = Frequency of Home Practice; In case of significant uncorrected *p*-values, corrected *p*-values are added between brackets. ⸷ Uncorrected effect becomes insignificant after correction for multiple testing following Benjamini-Hochberg procedure (1995).

**App Loggings Audio Files**

Besides the administration of ESM-questions, the You-Mind app logged usage data. For participants of the MT group, this included app starting times, visited audio and (introductory) videos (including duration of the interaction), and app error logging. As a measure of home practice, data on the visited audio files (i.e., guided mindfulness exercises), including duration of interaction, were analysed.

Approximately 38% (*n* = 36) of the participants of whom complete data were available (i.e., ESM and SRQs; *N* = 95), visited at least one audio file during the course of the trial. The average number of audio files visited in this group was 3.83 audio files (min. = 1.00, max. = 9). However, about one third only visited one audio file on the You-Mind app. Moreover, the average percentage of duration of interaction (i.e., audio file completion) for this group was about 22% (*M* = .219; *SD* = 0.375), which means that a considerable number of adolescents only partially listened to the visited audio file.

Subsequent analyses were conducted on the logged data of participants who listened to at least 50% of the visited audio file(s). For this subgroup (15.63%, *n* = 15), the average percentage of audio file completion was about 96% (*M* = .963, *SD* = 0.105). However, the mean number of audio files listened to for at least 50% remained rather low, namely 1.80 (min. = 1.00, max. = 4.00). Concerning the numbers of audio files listened to, 46.67% (*n* = 7), 33.33% (*n* = 5), 13.33% (*n* = 2), and 6.67% (*n* = 1) of the participants in this subgroup visited and listened for at least 50% to respectively 1, 2, 3, and 4 audio files.

Similar analyses were run for the sample of participants of whom SRQ data were available irrespective of ESM compliance (*N* = 221). Of this sample, 27.15% (*n* = 60) visited at least one audio file during the course of the trial, and 11.31% (*n* = 25) also listened to the audio files they visited for at least 50%. The mean number of audio files listened to for at least 50% was 2.48 audio files (min. = 1.00, max. = 14.00). Concerning the numbers of audio files listened to, 32% (*n* = 8), 40% (*n* = 10), 16% (*n* = 4), 8% (*n* = 2) and 4% (*n* = 1) of the participants in this subgroup at least partially listened to respectively 1, 2, 3, 4 and 14 audio files. Together these logged usage data strongly suggest that the majority of adolescents were not actively interacting with the audio files on the You-Mind app during the course of the trial. This finding is in line with the low average levels of reported home practice in this trial (see *Results*). It should be mentioned that the You mind app appeared to be incompatible with some particular types of smartphones (i.e., automatically pausing exercise when the smartphone entered sleep mode). This may have tempered participants motivation to use the guided exercises in the You-Mind app. Future studies should systematically collect data about the potential impact and scope of such technical issues. Nevertheless, given the overall low levels of practice, further research should collect information about adolescents’ needs and preferences. Such a process of co-designing may eventually increase the attractiveness of the material offered to support practice at home.

**Self-Experienced Positive and Negative Aspects Related to the MT**

At follow-up, as part of SRQs, participants were asked to mention three positive and three negative aspects about the MT they followed at school. Across all participants who completed the SRQs (irrespective of ESM compliance), a diverse range of answers was given.

Regarding positive aspects, participants most frequently mentioned that MT induced a sense of tranquillity or relaxation (*n* = 118; e.g., ‘It was relaxing’, ‘It helped me to calm down’, ‘It was soothing’). Also referring to a change at the emotional level, the stress-reducing effect or the experienced change in the way of responding towards negative emotions, and stress in particular, was often mentioned (*n* = 42; e.g., ‘less stress’, ‘learn how to deal with difficult feelings’, ‘stay calm in stressful situations’, ‘less thinking about things that could go wrong’, ‘You learn to accept things’, ‘It is okay not to feel good’). Besides, (increased) positive feelings appeared to be frequently experienced (*n* = 17, e.g., ‘I feel better’, ‘I am happier’, ‘It gives you a positive feeling’).

In addition to changes at the emotional level, also changes at the cognitive level were experienced. The following themes were mentioned: (a) being more attentive and aware (*n* = 91; e.g., ‘improved attention’, ‘becoming more aware of my body and environment’, ‘I became more aware of my thoughts and feelings’, ‘You learn more about yourself’, ‘You think about yourself’), (b) improved concentration (*n* = 10; e.g., ‘better concentration’, ‘more focus’, ‘I am calmer and I can focus better’), (c) peace of mind (*n* = 14; e.g., ‘It helped me to ‘clear’ my mind’, ‘It helped to calm down my mind’, ‘less thoughts’), and (d) change of perspective (*n* = 15; e.g., ‘You look at things from a different perspective.’, ‘Realising that a problem is actually smaller that you initially thought’). The following themes less clearly linked to either the cognitive or the emotional level, were mentioned: (a) it was experienced as a moment to pause (*n* = 10, e.g., ‘It distracts you for a moment from your busy life’, ‘You can temporarily forget your worries’), (b) it was a learning experience in itself (*n* = 22; e.g., ‘You learn a new skill’, ‘the experience in itself’, ‘something different’), (c) it improved sleep (*n* = 5; e.g., ‘I fall asleep more easily’, ‘It is good to sleep better’), (d) the group format was pleasurable (*n* = 8; e.g., ‘thinking about and discussing things together as a group’, ‘sociable’, ‘being together with the entire class group’), and (e) it was associated with an increased sense of control (*n* = 8, e.g., ‘I can control my hearth beat’, ‘I can better control my negative feelings’). Other positive aspects involved the guidance by the mindfulness trainer (*n* = 2) and the preference for particular mindfulness exercises (*n* = 5; e.g., ‘I particularly liked the body scan’). Finally, a non-MT related aspect frequently referred to was that the MT replaced actual courses/lessons (*n* = 25).

The negative aspects of the MT were roughly subdivided in experienced effects and practical aspects. First, some participants did not experience any changes, or experienced that changes did not impact their life in general (*n* = 12; e.g., ‘I do not have the impression that it has an impact on my daily life’, ‘It has not really helped me’, ‘I do not have the feeling that it was helpful for me’). Second, also unpleasant experiences related to the MT were mentioned (*n* = 22; e.g., ‘I become more aware of the negative thoughts or unpleasant physical experiences I have’, ‘I felt a bit disorientated and like I lost sense of time’, ‘more stress, anxiety, and sad feelings’, ‘short moment of sadness afterwards’). Third, some participants experienced no need for a MT, they were not interested in or experienced the MT as boring, or the MT did not really fit with them (*n* = 67; e.g., ‘I prefer something different to calm down’, ‘no need’, ‘a bit too wishy-washy for me’, ‘I do not like it’, ‘not my cup of tea’, ‘maybe a bit too young to think about yourself that much’, ‘a bit silly and boring’).

The following negative practical aspects were mentioned: (a) time investment or loss of time (*n* = 49; e.g., ‘It takes (too much) time’, ‘I prefer to invest my time in other things’, ‘It is a loss of time’), (b) duration of sessions and exercises (*n* = 37; e.g., ‘some exercises were too long’, ‘a bit long-winded’, ‘duration of sessions was too long’), (c) low level of physical activity (*n* = 17; e.g., ‘long time without moving’, ‘sitting still too long’), (d) being tiring or sleep-inducing (*n* = 25; e.g., ‘It makes me feel tired’, ‘Instead of calming me down, it was sleep-inducing’, ‘tiring’), (e) difficulties concentrating or being easily distracted (*n* = 22; e.g., ‘It was difficult to concentrate’, ‘difficult to stay concentrated’, ‘easily distracted’), (f) motivational issues (*n* = 21; e.g., ‘need a lot of patience’, ‘difficult to be motivated to do it’, ‘requires effort’), (g) difficulty (*n* = 16; e.g., ‘difficult to stay calm’, ‘sometimes it is difficult to start with an exercise’, ‘sometimes it is difficult’), and (h) too little variety (*n* = 9, e.g., ‘too much repetition’, ‘a lot of overlap in the exercises’, ‘too little variety in the exercises’). A few other, less frequently mentioned aspects referred to were: (a) the temporary nature of the exercises (*n* = 3, e.g., ‘You can stop for a moment, but everything around you keeps on going’), (b) the social aspect (*n* = 2; e.g., ‘Sometimes I was a bit reluctant to sharing feelings with my class group’), (c) the interference with the regular school curriculum (*n* = 2; e.g., ‘missed some classes’), (d) the suboptimal environment at school and group aspect (*n* = 11, ‘background noise’, ‘too busy environment’, ‘difficult with other people around you’, ‘stress about organisation of sessions’), and (e) too little interaction (*n* = 1).

**References**

Crane, R. S., Brewer, J., Feldman, C., Kabat-Zinn, J., Santorelli, S., Williams, J. M. G., & Kuyken, W. (2016). What defines mindfulness-based programs? The warp and the weft. *Psychological Medicine, 47*(6), 990–999. <https://doi.org/10.1017/S0033291716003317>

Santorelli, S. (2021). The essential spirit of mindfulness-based teaching. In R. S. Crane & G. M. Griffith (Eds.), *Essential Resources for Mindfulness Teachers* (1st edition, pp. 1-64). Routledge. https://doi-org/10.4324/9780429317880
